# Metatranscriptomic insights into microbial network modulation and pathogen dynamics underlying healing outcomes in non-surgical periodontal treatment

**DOI:** 10.1093/ismeco/ycag092

**Published:** 2026-04-11

**Authors:** Ryota Kobayashi, Takahiko Shiba, Takahiko Nagai, Keiji Komatsu, Shunsuke Matsumura, Takayasu Watanabe, Takashi Nemoto, Koki Takada, Yasuo Takeuchi, Takanori Iwata

**Affiliations:** Department of Periodontology, Graduate School of Medical and Dental Sciences, Institute of Science Tokyo, 1-5-45 Yushima, Bunkyo-ku, Tokyo 113-8510, Japan; Department of Periodontology, Graduate School of Medical and Dental Sciences, Institute of Science Tokyo, 1-5-45 Yushima, Bunkyo-ku, Tokyo 113-8510, Japan; Department of Periodontology, Graduate School of Medical and Dental Sciences, Institute of Science Tokyo, 1-5-45 Yushima, Bunkyo-ku, Tokyo 113-8510, Japan; Department of Lifetime Oral Health Care Sciences, Graduate School of Medical and Dental Sciences, Institute of Science Tokyo, 1-5-45 Yushima, Bunkyo-ku, Tokyo 113-8510, Japan; Department of Periodontology, Graduate School of Medical and Dental Sciences, Institute of Science Tokyo, 1-5-45 Yushima, Bunkyo-ku, Tokyo 113-8510, Japan; Department of Chemistry, Nihon University School of Dentistry, 1-8-13 Kanda-Surugadai, Chiyoda-ku, Tokyo 101-8310, Japan; Department of Periodontology, Graduate School of Medical and Dental Sciences, Institute of Science Tokyo, 1-5-45 Yushima, Bunkyo-ku, Tokyo 113-8510, Japan; Department of Periodontology, Graduate School of Medical and Dental Sciences, Institute of Science Tokyo, 1-5-45 Yushima, Bunkyo-ku, Tokyo 113-8510, Japan; Department of Lifetime Oral Health Care Sciences, Graduate School of Medical and Dental Sciences, Institute of Science Tokyo, 1-5-45 Yushima, Bunkyo-ku, Tokyo 113-8510, Japan; Department of Periodontology, Graduate School of Medical and Dental Sciences, Institute of Science Tokyo, 1-5-45 Yushima, Bunkyo-ku, Tokyo 113-8510, Japan

**Keywords:** metatranscriptomics, periodontitis, gingivitis, subgingival plaque, oral microbiome, bacterial network, non-surgical periodontal treatment

## Abstract

Gingivitis and periodontitis are caused by oral microbiome dysbiosis. Post-treatment alterations in bacterial community structure are uncharacterized in situ, including how these alterations may differ between resolved and unresolved disease states. Understanding these treatment-induced microbial shifts and identifying prognostic markers in situ associated with favorable or unfavorable outcomes are crucial for developing diagnostic tools and refining therapeutic strategies. Therefore, we performed metatranscriptomic analysis on subgingival plaque samples from the anterior teeth of individuals, including healthy, gingivitis, and periodontitis sites, before and after non-surgical treatment in 28 patients. We revealed a new bacteriological characteristic of periodontitis, where periodontal pathogens emerge within the bacterial network alongside excessive and skewed associations among bacterial taxa, such as those in the *Streptococcus* and *Actinomyces* genera. Furthermore, these imbalances were found improvable through non-surgical treatment. However, even in clinically resolved gingivitis or periodontitis, the bacterial networks did not fully revert to the state observed in healthy sites. This was due to the persistence of periodontal pathogens, absent in the networks at healthy sites. By comparing groups in which periodontitis resolved and those in which it did not, specific bacterial taxa, such as *Neisseria elongata* and *Rothia aeria*, were suggested to play a role in the periodontitis healing process, while increases in genes related to glycine degradation and bacterial adhesion, including glycine dehydrogenase β-subunit and cleaved adhesin domain were implicated in inhibiting the healing process. These findings provide insights for the development of treatment strategies targeting specific bacteria and functional genes involved in the resolution of periodontitis.

## Introduction

Periodontitis, an irreversible disease characterized by gingival inflammation and alveolar bone resorption around the teeth, is an infectious disease caused by dysbiosis of the oral microbiome [1–4], and its global prevalence is remarkably high [5, 6]. Moreover, the disease is associated with several systemic diseases [7–12]. Gingivitis, which is considered a precursor to periodontitis, is a reversible disease that causes gingival inflammation [13]. Gingivitis, an intermediate state between health and periodontitis, serves as a comparative reference for understanding how the bacterial community structure changes during both periodontitis progression and healing [14, 15]. These clinical conditions are reflected as oral microbiome dysbiosis, and etiological studies indicate that a specific bacterial species is associated with this dysbiosis [16–18]. The keystone pathogen is a microorganism that has a disproportionately large effect on its environment relative to its abundance; for example, *Porphyromonas gingivalis*, a representative keystone pathogen in periodontitis, is a member of the red complex, which is a group of strongly periodontopathogenic bacteria associated with periodontal disease, together with *Tannerella forsythia* and *Treponema denticola*. The identification of keystone pathogens and the bacteria influenced by them is important for diagnosis and treatment of these oral conditions [19, 20]. The identification of key pathogens requires comprehensively capturing only live bacterial taxa, including those that are difficult to culture, using sequencing and bioinformatics analysis.

Metatranscriptomic analysis enables comprehensive understanding of the pathogenesis of periodontitis in situ, as it can detect live bacterial species, active functional genes, and active metabolic pathways [1, 3, 21]. Using metatranscriptomic analysis, Nemoto *et al*. [1] showed that the bacterial co-occurrence network at gingivitis sites was in a transitional state from healthy to periodontitis. A study that performed metatranscriptomic analysis of both progressive and non-progressive sites within the same individuals with periodontitis identified differences in metabolic activities between these sites [22].

Non-surgical treatment involving mechanical debridement of both supra-and subgingival plaque and calculus is fundamental to the management of periodontal disease [23]. Diao *et al*. [24] performed 16S ribosomal RNA (rRNA) sequencing to investigate the effect of non-surgical treatment on the oral microbiome in patients with periodontitis; they found that the bacterial composition of patients could not revert to a healthy state even after treatment. Although the effects of non-surgical treatment on microbiome in periodontitis have been investigated, only a few studies have used metatranscriptomic analyses. Furthermore, whether the microbiome in periodontitis or gingivitis sites within the same patient shifts towards a healthy state with no history of periodontal disease after treatment or transitions into a state prone to recurrence remains uninvestigated. Notably, this remains unclear even within the context of the same patient and the same tooth types.

This study hypothesizes that treatment interventions influence the ecological dynamics of the oral microbiome in diseased sites but that persistent bacterial groups and networks hinder the re-establishment of microbial homeostasis. Therefore, in this study, we aimed to clarify the bacteriological characteristics and treatment-induced changes in subgingival plaque under healthy, gingivitis, and periodontitis conditions by prospectively comparing samples obtained from the same patients and only anterior teeth, thereby eliminating inter- and intra-individual variability. Additionally, by grouping sites based on treatment outcomes, we sought to investigate the bacteriological features that contribute to differences in treatment efficacy, even in cases that appear to present the same clinical condition of periodontitis, with the goal of providing insights toward the development of an early prognostic method for treatment outcomes.

## Materials and methods

### Ethical statement

This research was conducted according to the Ethical Guidelines for Clinical Studies (Ministry of Health, Labor, and Welfare notification number 415, 2008). Approval was obtained from the Ethics Committee of the Tokyo Medical and Dental University (currently Institute of Science Tokyo), Tokyo, Japan (D2015–535). Written informed consent was obtained from all participants before their participation in the study. This study adhered to the principles outlined in the Declaration of Helsinki, which was amended in 2013.

### Study population and non-surgical treatment

We included patients who underwent non-surgical periodontal treatment at the Tokyo Medical and Dental University Hospital (currently Institute of Science Tokyo Hospital). Post-treatment samples were newly collected from a subset of participants included in our previous metatranscriptomic study by Nemoto *et al*. [1] (*n* = 11). In addition, pre- and post-treatment samples were collected from newly enrolled participants (*n* = 17). Accordingly, the present study analysed a total of 28 individuals for whom paired pre- and post-treatment samples were available. Based on the 2017 World Workshop classification, all participants were diagnosed with Stage III periodontitis at the patient level, with 18 patients classified as Grade B and 10 patients as Grade C (Table S1). Periodontal probing depth (PPD) was assessed at six sites per tooth. These patients had healthy (PPD ≤ 3 mm without bleeding on probing [BOP]), gingivitis (PPD ≤ 3 mm with BOP), and periodontitis (PPD ≥ 4 mm with BOP, clinical attachment loss, and radiographic bone loss [RBL]) sites in maxillary or mandibular anterior teeth [25, 26]. All patients underwent pre-treatment sampling, followed by non-surgical periodontal treatment. Subgingival plaque samples were collected from the deepest pockets at gingivitis and periodontitis sites. Healthy site samples were collected from sites located at least one tooth away from the gingivitis and periodontitis sites, with care taken to maximize positional separation where possible. Previous studies have reported that the bacterial community differs between anterior and posterior teeth [27]. Therefore, to minimize intra-oral heterogeneity arising from anatomical and environmental factors that may influence the bacterial community—such as saliva flow and tongue movement—sampling was limited to anterior teeth. Ten sterilized paper points (#30, a relatively narrow size to minimize trauma or bleeding) were gently inserted into each pocket up to the base of the sulcus for 60 seconds. At healthy sites, the paper points were carefully inserted only until slight resistance was felt, using gentle manipulation to avoid trauma or bleeding during sampling. None of the participants received systemic antibiotics or anti-inflammatory agents from 3 months before the baseline examination until post-treatment sampling. The participants had no systemic diseases and no history of smoking [1, 3, 28]. The patients received non-surgical treatment, which comprised oral hygiene instructions, scaling and root planning of all sites, and occlusal adjustment when necessary. Subgingival instrumentation was performed on all examined teeth, including clinically healthy sites. At healthy sites, instrumentation was conducted within a range that did not compromise the existing periodontal attachment. Post-treatment sampling was conducted at least three months after treatment completion.

### Sample collection and library preparation

The supragingival plaque was removed using sterile cotton pellets, and ten sterilized paper points were inserted into the pocket for 60 seconds. The points were collected in a sterilized tube, immediately immersed in liquid nitrogen, and then stored at −80°C until RNA extraction.

RNA was extracted, polyadenylated, and reverse-transcribed into complementary DNA (cDNA) using the same methods in our previous paper [1] and Text S1. Metatranscriptome sequencing libraries were constructed using a Nextera XT DNA Library Preparation Kit (Illumina, San Diego, CA, USA). The prepared samples were pooled, and 300-base pair (bp) paired-end reads were generated using the Illumina MiSeq system platform. Details of nucleic acid quality control at each step and the products used are provided in Text S1. Samples were processed under strict low-temperature conditions, and RNA concentration and integrity were carefully assessed using a Quantus fluorometer (Promega, Madison, WI, USA) and an Agilent 2100 Bioanalyzer system (Agilent Technologies, Santa Clara, CA, USA) prior to library preparation. Samples with an RNA integrity number (RIN) of ≥5.0 were considered eligible for metatranscriptomic sequencing.

### Processing and analysing Illumina sequencing data

The sequencing data obtained in this study were analysed together with data downloaded from the DNA Data Bank of Japan (DDBJ) (DRA011737), which were derived from 21 patients before non-surgical treatment in a previous study. The analytical tools and detailed procedures used are provided in Text S1; the key points are summarized below. To ensure comparability across datasets, experimental procedures were standardized across studies, and batch effects [29] associated with data derived from the previous and current studies were addressed using the Conditional Quantile Regression method implemented in the R package ConQuR (version 2.0) [30], as described in Text S1, prior to downstream analyses. For taxonomic analysis, reconstructed 16S rRNA genes (rc-rRNA) assembled from paired-end reads were obtained using the expectation maximization iterative reconstruction of genes from the environment (EMIRGE) pipeline [31]. We classified the representative sequence of each rc-rRNA operational taxonomic unit (OTU) as similar to that in the Human Oral Microbiome Database (HOMD), version 13.2 [32], using the Basic Local Alignment Search Tool N (BLASTN) [33, 34]. The number of OTUs and Shannon index were used to estimate the α diversity indices. The abundance of all rc-rRNA OTUs was normalized using centered log-ratio (CLR) values [35]. Subsequently, community diversity was compared using permutational multivariate analysis of variance (PERMANOVA) and visualized using principal component analysis (PCA), both based on the Aitchison distance. Based on previous studies [3, 36], the mRNA OTUs were used to identify putative virulence factors using Basic Local Alignment Search Tool X (BLASTX) [37] against the National Center for Biotechnology Information non-redundant (NCBI nr) protein database (as of October 31, 2014), Virulence Factors of Pathogenic Bacteria database (VFDB) (as of February 9, 2015), and Microbial Virulence Database (MvirDB) (as of October 9, 2014), and protein function profiles were obtained.

The abundance values of all mRNA OTUs were normalized by conversion to transcripts per million (TPM) to account for differences in gene length and library size. The Bray–Curtis distance was used for PERMANOVA and principal coordinate analysis (PCoA). Additionally, differential abundance analyses of rc-rRNA-based bacterial taxa and mRNA-derived functional features were performed using the ANCOM-BC2 method implemented in the R package ANCOMBC (version 2.4.0) [38]. As a complementary analysis, exploratory odds ratio–based analyses were performed using univariate logistic regression in the R stats package (version 4.3.1) [39], with CLR–transformed rc-rRNA–based bacterial abundance values as explanatory variables and post-treatment healing outcomes as the dependent variable.

Only the taxa identified in both rc-rRNA and mRNA OTUs were included for further analyses, and these taxa were defined as viable taxa with in situ functions (VTiFs) [3, 36]. These VTiFs were used to construct network structures. Correlation coefficients were calculated using the Sparse Correlations for Compositional data (SparCC) software [40] based on mRNA taxonomic abundances. Taxon pairs with SparCC values of ≥0.85 and ≤ −0.8 were regarded as positive and negative relationships, respectively. Only taxon pairs with significance identified using the Benjamini–Hochberg (BH) method (*q* < 0.05) were visualized using Cytoscape software, version 3.10.2 [41]. The number of nodes and edges and the values of network density, clustering coefficient, and network centralization were also calculated for each group using Cytoscape. Nodes represent VTiFs (bacterial taxa) that participate in at least one significant SparCC association under the predefined correlation and significance thresholds, and node size reflects abundance based on mRNA read counts.

### Statistical analysis

For the statistics of clinical parameters and α diversities, R-based analyses were performed using R (version 4.3.1; R Foundation for Statistical Computing, Vienna, Austria). Friedman and Kruskal–Wallis tests were used for paired and unpaired data, respectively. Dunn’s multiple comparison test was used to identify significantly different groups. The Wilcoxon matched-pairs test was used to test for significant differences between groups. The tests were conducted using the statistical software GraphPad Prism (Version 10.1.1; San Diego, CA, USA). PERMANOVA was used to compare the similarity of the taxonomic and functional profiles of each group using adonis2 in the R package vegan (version 2.6.4) [42] with 999 permutations. This was followed by post hoc pairwise comparisons using the Bonferroni method with the R package pairwiseAdonis (version 0.4.1) [43]. To determine the significance of the abundance of differentially abundant bacterial taxa (DAT) and differentially expressed genes (DEGs), BH method implemented in the R stats package (version 4.3.1) was used, with *q* < 0.05 considered statistically significant. Sensitivity analysis implemented in ANCOM-BC2 was used to assess the reliability of the detected taxa and functional features, including the impact of pseudo-count addition on zero-inflated data.

## Results

### Study population and clinical parameters

We collected baseline data from healthy (H1), gingivitis (G1), and periodontitis (P1) sites of 28 patients, where the suffix “1” indicates pre-treatment status. After non-surgical periodontal treatment, all H1 sites remained healthy (H2), and all G1 sites showed resolution of bleeding on probing and were reclassified as healthy sites (G2). Periodontitis sites at baseline (P1) were categorized at re-evaluation into diseased periodontitis sites (DP2), defined by probing pocket depth (PPD) ≥ 4 mm with persistent bleeding on probing (BOP), or resolved periodontitis sites (RP2), defined by the absence of BOP. Accordingly, P1 sites were retrospectively classified into diseased periodontitis (DP1) and resolved periodontitis (RP1) groups based on post-treatment clinical outcomes. Owing to insufficient sequencing read counts, two G1 samples and one P1 sample were excluded from analyses involving pre-treatment data. Therefore, baseline cross-sectional comparisons among H1, G1, and P1 were conducted using data from the remaining 25 patients. For the longitudinal pre- and post-treatment analyses, only samples with insufficient sequencing read counts were excluded, 28, 26, 13, and 14 samples from the H, G, DP, and RP groups, respectively, were analysed. In contrast, no sample loss occurred at the post-treatment time point, and post-treatment cross-sectional analyses were therefore conducted using all 28 participants.

The interval from completion of non-surgical periodontal treatment to post-treatment sampling did not differ significantly between the resolved and diseased periodontitis groups, indicating minimal influence of follow-up timing on the group comparisons (Table S1). Table 1 summarizes the longitudinal and cross-sectional clinical parameters at pre- and post-treatment across the healthy, gingivitis, diseased periodontitis, and resolved periodontitis groups. Table S2a summarizes the overall pre- and post-treatment changes in the proportion of sites with PPD ≥ 4 mm in the anterior and posterior regions, while Table S2b presents the same analysis stratified by anterior-site resolution status into the DP and RP groups. Significant reductions were observed in both anterior and posterior regions after treatment (Table S2a). Similar reductions were observed in both the DP and RP groups (Table S2b), although cases classified as resolved at the anterior sites were not necessarily similarly resolved in the posterior region. We observed no significant differences in the PPD and RBL before treatment between the DP1 and RP1 groups (adjusted *P* > .99 both). However, we observed significantly reduced PPDs after treatment in G, DP, and RP groups. Additionally, the DP2 and RP2 groups demonstrated differences in PPD after treatment (adjusted *P* = .02). All H2, G2, and RP2 sites were BOP-negative. In contrast, the percentage of BOP in DP2 was 100% (Table 1). The ecological continuum across periodontal conditions and the outcome-dependent remodeling of periodontitis-associated bacterial networks before and after treatment are summarized in Fig. S1a and b, respectively.

**Table 1:** The clinical parameters for individuals sampled at pre- and post-treatment for each group^x^.

Site		Healthy	Gingivitis	Periodontitis	
				Diseased group	Resolved group
PPD (mm)	Pre-treatment	H1: 2.4 ± 0.6^ab^	G1: 2.7 ± 0.5^cd^	DP1: 6.3 ± 1.4^ac^	RP1: 5.2 ± 1.8^bd^
	Post-treatment	H2: 2.3 ± 0.5^a^	G2: 2.4 ± 0.5^c^	DP2: 5.7 ± 2.1^ace^	RP2: 3.1 ± 1.2^e^
	*p*-value (within groups)	> 0.99	0.02	0.008	0.0002
RBL (mm)	Pre-treatment	H1: 2.81 ± 1.01^ab^	G1: 2.96 ± 1.08^cd^	DP1: 6.69 ± 2.30^ac^	RP1: 6.28 ± 2.34^bd^
	Post-treatment	H2: 2.71 ± 0.85^ab^	G2: 2.78 ± 1.10^cd^	DP2: 6.28 ± 2.30^ac^	RP2: 4.16 ± 1.44^bd^
	*p*-value (within groups)	0.48	0.03	0.41	0.0001
BOP (% of sites)	Pre-treatment	0	100	100	100
	Post-treatment	0	0	100	0

^x^Values represent means ± standard deviation. H, G, DP, and RP indicate healthy gingival tissue, gingivitis, diseased periodontitis, and resolved periodontitis, respectively. The number 1 after each group name represents pre-treatment and the number 2 represents post-treatment. Only samples with sufficient sequencing reads were used, resulting in 28, 26, 13, and 14 sites compared longitudinally and cross-sectionally in the H, G, DP, and RP groups, respectively. Significant differences were observed between the groups marked with the same symbol (adjusted *P* < .05). PPD: probing pocket depth; RBL: radiographic bone loss; BOP: bleeding on probing. ^a^Significant differences between H and DP. ^b^Significant differences between H and RP. ^c^Significant differences between groups G and DP. ^d^Significant differences between groups G and RP. ^e^Significant differences between the DP and RP groups.

### Diversity analysis in each bacterial community

**Figure 1:**
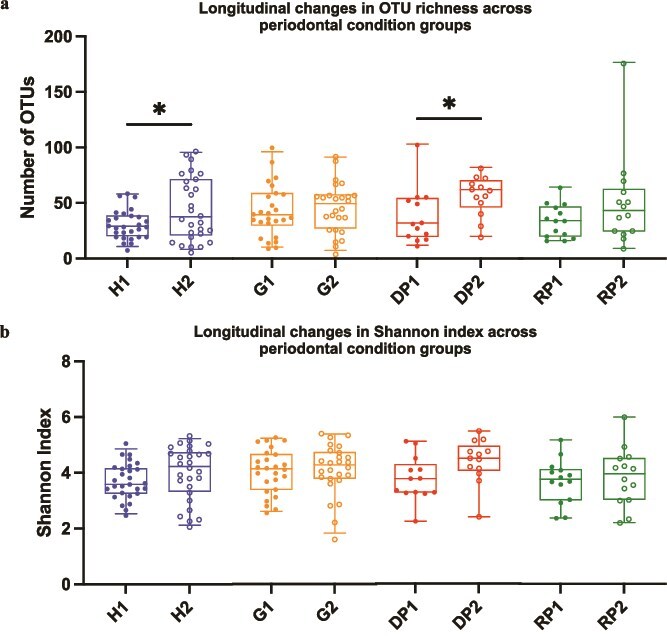
Longitudinal changes in α diversity following non-surgical periodontal therapy. The number of operational taxonomic units (OTUs) (a) and the Shannon index (b) are shown for all groups at pre- and post-treatment. H, G, DP, and RP indicate healthy, gingivitis, diseased periodontitis, and resolved periodontitis sites, respectively. The suffixes “1” and “2” denote pre-treatment and post-treatment status. Boxplots represent the interquartile range (25th–75th percentiles), with the median shown as a center line and whiskers indicating the 95% confidence interval. Although all groups are displayed together for visual comparison, statistical analyses in this figure focus exclusively on longitudinal (paired) comparisons within each group (H1 vs H2, G1 vs G2, DP1 vs DP2, and RP1 vs RP2). Cross-sectional multi-group analyses are presented separately in Fig. S3. Statistical significance for within-group pre- and post-treatment comparisons was tested using the Wilcoxon matched-pairs test and is indicated by ^*^ (*P* < .05).

Sequence read information is summarized in Tables S3 and S4, whereas Fig. S2 shows rarefaction curves generated from EMIRGE-derived rc-rRNA OTU abundance rather than from total library size. For α diversity analysis, the number of OTUs assigned to rc-rRNA obtained using the EMIRGE pipeline [31] was first evaluated (Fig. 1a–d). We observed a significant increase in the number of OTUs in H and DP groups before and after treatment despite no change in clinical diagnosis in either group (Fig. 1a and c). Although the number of OTUs did not differ within G sites or within RP sites before and after treatment, we observed an increasing trend (Fig. 1b and d). We observed no significant differences in the number of OTUs in the cross-sectional comparisons among different periodontal tissue conditions, either before or after treatment (Fig. S3a and b). In addition, we observed no significant differences in the Shannon index within or between any of the groups before and after treatment (Fig. S3c and d).

**Figure 2:**
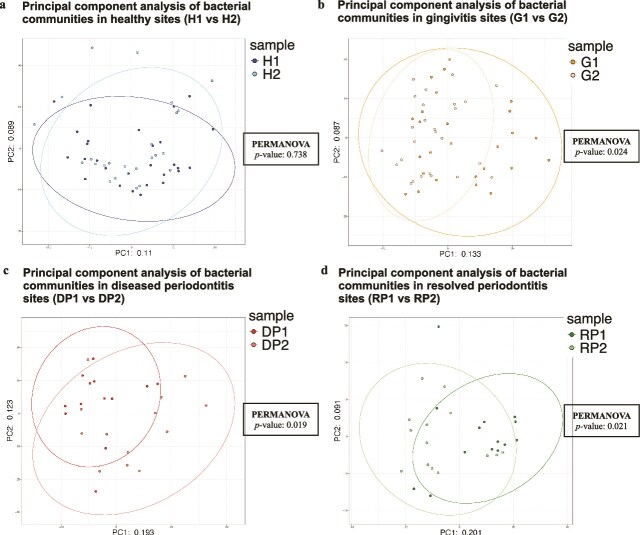
Β diversity of bacterial communities in pre- and post-treatment samples. Β diversity was assessed using principal component analysis (PCA) based on Aitchison distance calculated from centered log-ratio (CLR)-normalized rc-rRNA operational taxonomic unit (OTU) abundances mapped to the human Oral microbiome database (HOMD). Each point represents one sample; darker colors indicate pre-treatment samples and lighter colors indicate post-treatment samples. Ellipses indicate 95% confidence intervals. Panels show longitudinal (paired) comparisons within each group: (a) healthy (H), (b) gingivitis (G), (c) diseased periodontitis (DP), and (d) resolved periodontitis (RP). The numbers “1” and “2” denote pre-treatment and post-treatment status, respectively. The numbers of paired samples analysed were H: *n* = 28, G: *n* = 26, DP: *n* = 13, and RP: *n* = 14. Differences in community composition between pre- and post-treatment samples within each group were tested by permutational multivariate analysis of variance (PERMANOVA) using Aitchison distance; *P*-values are shown in each panel. Abbreviations: PCA, principal component analysis; PERMANOVA, permutational multivariate analysis of variance; HOMD, human Oral microbiome database; CLR, centered log-ratio; OTU, operational taxonomic unit.

Regarding β diversity, longitudinal bacteriological comparisons before and after treatment revealed significant differences in the G, DP, and RP groups PERMANOVA, *P* = .024, *P* = .019, *P* = .021, respectively, but not in the H group (*P* = .738) (Fig. 2a–d). In a cross-sectional comparison of the four pre-treatment groups, PCA showed that the distributions of DP1 and RP1 overlapped more than those between other group, with no statistically significant differences between them. However, we observed significant differences in the β diversities of H1 and DP1, H1 and RP1, and G1 and DP1 (PERMANOVA, adjusted *P* = .006, adjusted *P* = .006, and adjusted *P* = .042, respectively) (Fig. S3e). Conversely, the PCA plot indicated that the distributions of H2, G2, DP2, and RP2 were clustered together. We observed no significant differences in β diversities between H2, RP2, and DP2 after treatment, and an l difference between β diversities of G2 and DP2 (Fig. S3f).

### Differential abundance analysis based on bacterial abundance

**Figure 3:**
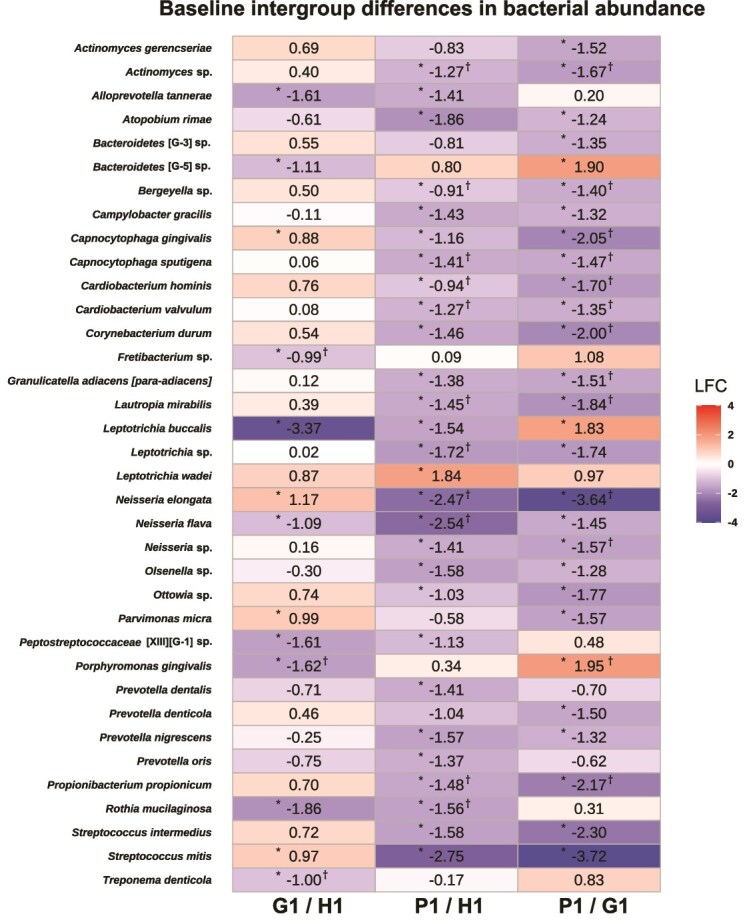
Baseline intergroup differences in bacterial abundance based on human Oral microbiome database (HOMD) annotation. Differential abundance analysis was performed using ANCOM-BC2 based on bacterial abundance data from pre-treatment samples only (*N* = 25), after excluding samples with insufficient sequencing depth. Comparisons were conducted among healthy (H1), gingivitis (G1), and periodontitis (P1) sites at baseline. This heatmap summarizes the multiple pairwise comparisons generated within a single ANCOM-BC2 analysis including all three baseline groups. The column labels indicate the direction of the contrast: G1/H1 represents differences in gingivitis sites relative to healthy sites, P1/H1 represents differences in periodontitis sites relative to healthy sites, and P1/G1 represents differences in periodontitis sites relative to gingivitis sites. Positive log fold change (LFC) values indicate higher abundance in the numerator group, whereas negative values indicate higher abundance in the denominator group. Only taxa that showed statistically significant differences in at least one pairwise comparison by ANCOM-BC2 are displayed (Benjamini–Hochberg-adjusted *q* < 0.05). For these displayed taxa, LFC values are shown for all three pairwise comparisons, including comparisons that did not reach statistical significance. Heatmap cells display LFC values, and color intensity indicates the magnitude and direction of change. Asterisks (^*^) indicate statistically significant pairwise comparisons, and taxa that additionally passed the ANCOM-BC2 sensitivity analysis are marked with a dagger symbol (†). To improve interpretability, LFC values are capped within the displayed color scale. Abbreviations: H, healthy; G, gingivitis; P, periodontitis; suffix “1” indicates pre-treatment status; LFC, log fold change; ANCOM-BC2, analysis of compositions of microbiomes with bias correction, version 2.

**Figure 4:**
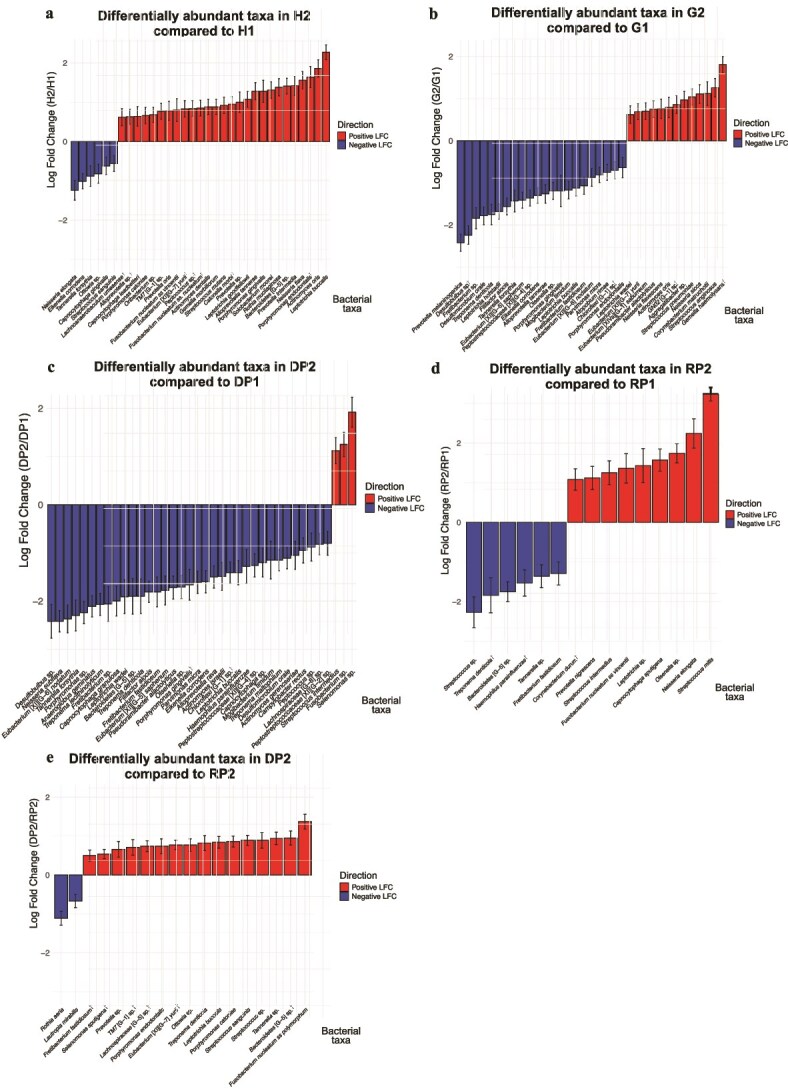
Differentially abundant taxa (DAT) identified by ANCOM-BC2 in pre- and post-treatment comparisons. Only taxa meeting the multiple-testing–adjusted significance threshold (Benjamini–Hochberg–adjusted *q* < 0.05) are displayed. Taxa showing increased abundance after treatment are plotted above zero, whereas those showing decreased abundance are plotted below zero. Bar height represents the log fold change (LFC), and whiskers indicate ±1 standard error. Taxa that additionally passed the ANCOM-BC2 sensitivity analysis are indicated by a dagger symbol (†) to the right of the taxon name, denoting robustness of the result rather than statistical significance. The four groups are defined as follows: H, healthy; G, gingivitis; DP, diseased periodontitis; and RP, resolved periodontitis. The numbers “1” and “2” indicate pre- and post-treatment, respectively. Panels (a–d) show within-group longitudinal comparisons (H2 vs H1, *n* = 28; G2 vs G1, *n* = 26; DP2 vs DP1, *n* = 13; RP2 vs RP1, *n* = 14). Panel (e) shows the post-treatment comparison between DP2 and RP2 (*n* = 14 vs 14).

The OTUs were assigned to bacterial taxa based on the HOMD to obtain bacterial composition data for each group (Fig. S4). DAT were evaluated by ANCOM-BC2 [38]. In the baseline intergroup comparison (Fig. 3), several taxa exhibited distinct abundance patterns along disease progression. *Capnocytophaga gingivalis* showed an increased abundance in G1 compared with H1. In contrast, *Neisseria elongata* was significantly decreased in P1 compared with H1. Notably, in the comparison between P1 and G1, *P. gingivalis* and *Bacteroidetes* [G-5] sp. were significantly increased in P1 compared with G1, whereas *N. elongata* was further decreased. These taxa did not show an increase in the G1/H1 comparison but became enriched only when P1 was compared with G1. In addition to the baseline intergroup comparisons, longitudinal comparisons were also performed to assess treatment-associated changes in bacterial abundance. The numbers of DAT detected in the H, G, DP, and RP groups based on HOMD were 35, 33, 38, and 15, respectively (adjusted *P* < .05, Fig. 4a–d). In addition, to facilitate comparison of shared and group-specific bacterial changes across the four groups, the DAT identified in H, G, DP, and RP were integrated and summarized in Fig. S5. In the H group, despite no significant difference in β diversity, we observed multiple DAT, along with decreased proportions of facultative anaerobes and *T. forsythia*, an obligate anaerobe and a member of the red complex, at post-treatment compared with pre-treatment. Interestingly, by contrast, *P. gingivalis*, another member of the red complex, increased in abundance after treatment (Fig. 4a). To elucidate the details of the unexpected increase in *P. gingivalis* after treatment, we evaluated DAT, identified by comparing H1 and H2, separately for individuals at sites DP2 and RP2. The results indicated an increase in *P. gingivalis* at H2 sites in individuals with DP2 sites and a decrease in *T. forsythia* at H2 sites in individuals with RP2 sites (Fig. S6a and b). In the G group, the majority of bacteria that decreased after treatment were anaerobic, including the red complex (Fig. 4b). We also compared DAT detection between DP2 and RP2 individuals in the G group, identified by comparing G1 and G2. Individuals with RP2 demonstrated reduced proportions of several obligate anaerobes, including *T. forsythia, P. gingivalis* and *Fusobacterium nucleatum* subsp. *vincentii* at post-treatment, whereas individuals with DP2 demonstrated a smaller reduction in the proportions of obligate anaerobes, such as *T. denticola*, post-treatment than the RP2 group (Fig. S6c and d). In the DP group, the number of increased anaerobic bacteria among the DAT was only 3 bacterial taxa including *T. denticola* (Fig. 4c), whereas in the RP group, multiple aerobic bacteria, including *Streptococcus mitis* and *N. elongata*, increased (Fig. 4d). Additionally, to focus on the bacterial taxa related to the prognosis and healing of periodontitis, we compared the DAT between the DP1 and RP1 groups and between the DP2 and RP2 groups. We found no DAT between the DP1 and RP1 groups; however, 18 DAT were in between the DP2 and RP2 groups. Compared with the RP2 group, the DP2 group demonstrated a significantly lower abundance of *Rothia aeria* and *Lautropia mirabilis*, and significantly higher abundances of taxa such as *Bacteroidetes* [G-5] sp. and *Porphyromonas endodontalis* (Fig. 4e). To further explore whether these taxa were associated with healing outcomes at the sample level, we additionally performed exploratory odds ratio–based analyses using abundance values. Although no definitive associations were identified, several taxa that were enriched in DP2 sites, including *Bacteroidetes* [G-5] sp. and *P. endodontalis*, showed a tendency toward higher odds of being classified as diseased sites (Table S5).

### Differential abundance analysis based on functional gene abundance

Putative mRNA sequences were annotated as functional genes with NCBI nr protein database, VFDB, and MvirDB. PCoA for the comparison of functional gene composition, which was detected in NCBI nr, showed significant differences between pre- and post-treatment in all groups (Fig. S7a–d). In the pre-treatment cross-sectional analyses, we observed no significant differences between the H1 and G1 groups or between the DP1 and RP1 groups (Fig. S7e). In contrast, there were significant differences between the H1 and DP1, H1 and RP1, G1 and DP1, and G1 and RP1 groups. After treatment, there were no significant differences between any of the groups (Fig. S7f). The results obtained from the VFDB and MvirDB showed a tendency similar to that of NCBI nr (Figs S8–S10).

**Figure 5:**
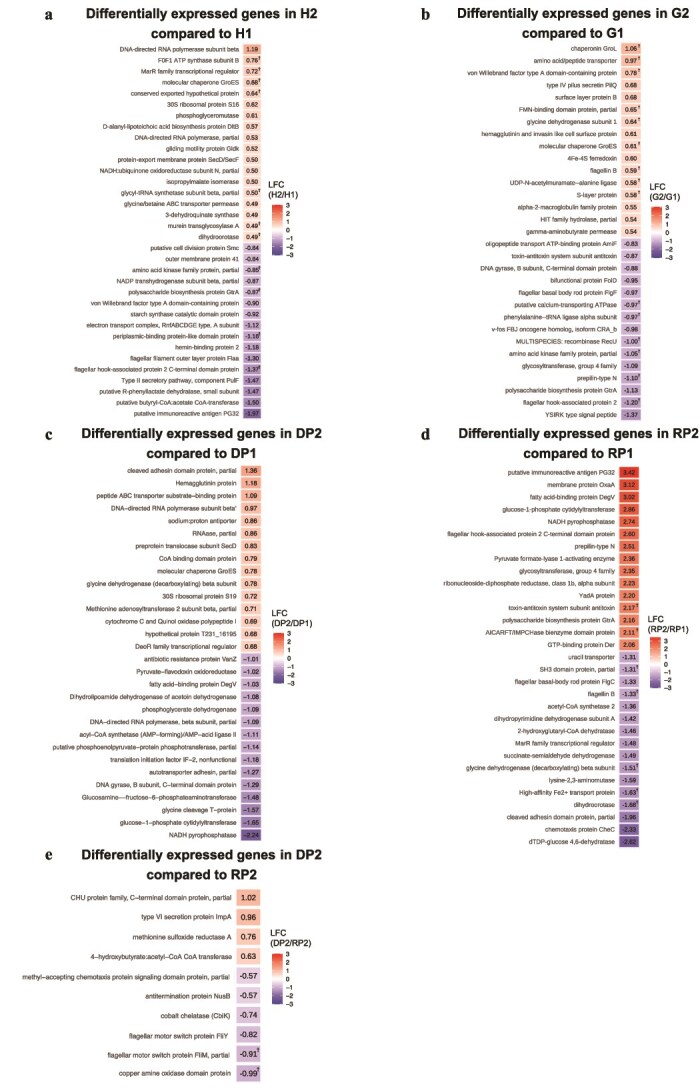
Heatmaps of differentially expressed genes (DEGs) with significant expression changes in each group based on the National Center for Biotechnology Information non-redundant (NCBI nr) protein database. DEGs were identified using ANCOM-BC2 with Benjamini–Hochberg (BH) correction, and genes with *q* < 0.05 were considered statistically significant. Only statistically significant DEGs are shown in the heatmaps. The numbers in the heatmaps indicate log fold change (LFC) values, and DEGs that passed sensitivity analysis are marked with a dagger symbol (†). H, G, DP, and RP indicate healthy, gingivitis, diseased periodontitis, and resolved periodontitis sites, respectively. The numbers “1” and “2” denote pre-treatment and post-treatment status, respectively. Panels (a–d) show within-group longitudinal comparisons between pre- and post-treatment (H2 vs H1, *n* = 28; G2 vs G1, *n* = 26; DP2 vs DP1, *n* = 13; RP2 vs RP1, *n* = 14). Panel (e) shows the post-treatment comparison between DP2 and RP2 (*n* = 14 vs 14). Abbreviations: DEG, differentially expressed gene; LFC, log fold change; ANCOM-BC2, analysis of compositions of microbiomes with bias correction, version 2; BH, Benjamini–Hochberg; NCBI nr, National Center for biotechnology information non-redundant protein database.

DEGs were evaluated using R package ANCOM-BC2 [38] (Fig. 5, Figs S11–S13). In the NCBI nr protein database, when comparing pre- and post-treatment, the numbers of significantly enriched DEGs were 881, 644, 274, and 456 in the H, G, DP, and RP groups, respectively. The top 15 DEGs with the largest absolute log-fold changes were selected and are shown in Fig. 5. Among the selected DEGs, genes encoding amino acid kinase family proteins and polysaccharide biosynthesis proteins were downregulated after treatment in both the H and G groups (Fig. 5a and b). Five DEGs were commonly detected in the DP and RP groups: genes encoding fatty acid-binding protein DegV, glucose-1-phosphate cytidylyltransferase, nicotinamide adenine dinucleotide hydrogen (NADH) pyrophosphatase, glycine dehydrogenase (decarboxylating) beta subunit, and partial cleavage of the adhesin domain protein (Fig. 5c and d). The expression of the first three DEGs increased after treatment in the RP group and decreased after treatment in the DP group, whereas the expression of the latter two DEGs decreased after treatment in the RP group and increased after treatment in the DP group. We observed that no DEGs differed between DP1 and RP1 in any of the reference databases. In contrast, 10 and 12 DEGs identified in the NCBI nr protein and MvirDB differed between DP2 and RP2, respectively (Fig. 5e, Fig. S13). In the NCBI nr database, genes encoding the C-terminal domain protein and type VI secretion protein were upregulated in DP2 compared to RP2. Similarly, in MvirDB, genes encoding endopeptidase and SubE were more upregulated in DP2 than in RP2.

### Bacterial correlation network analysis

**Figure 6:**
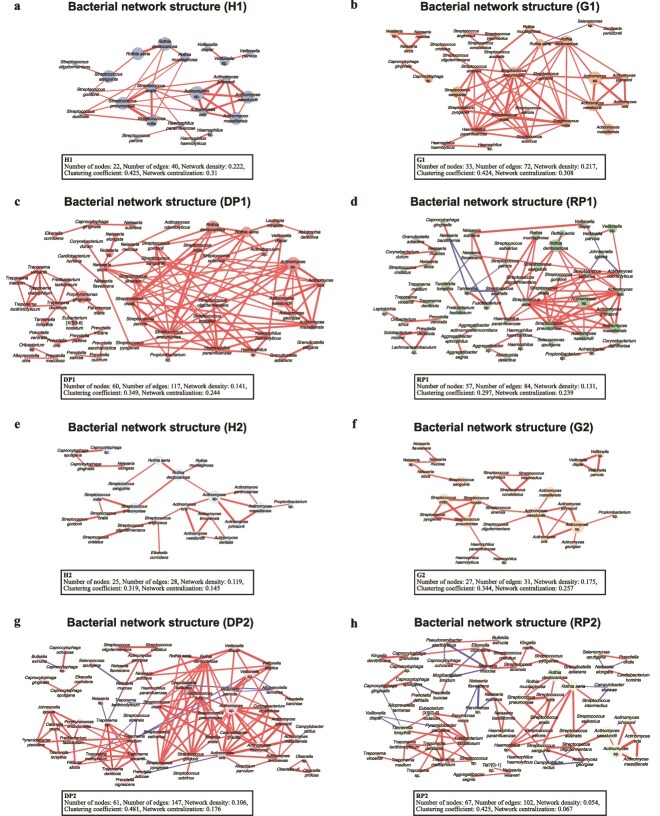
Network analyses of subgingival bacterial communities in pre- and post-treatment samples for each group. The numbers of samples analysed in each group were as follows: H1, *n* = 28; G1, *n* = 26; DP1, *n* = 13; RP1, *n* = 14; H2, *n* = 28; G2, *n* = 28; DP2, *n* = 14; RP2, *n* = 14. Group abbreviations are defined as follows: H, healthy sites; G, gingivitis sites; DP, diseased periodontitis sites; RP, resolved periodontitis sites. The suffixes “1” and “2” indicate pre-treatment and post-treatment status, respectively. Networks were constructed using viable taxa with in situ functions (VTiFs) identified in both rc-rRNA and mRNA profiles. Pairwise correlations were calculated using sparse correlations for compositional data (SparCC) based on mRNA-derived taxonomic abundances. Taxon pairs with SparCC correlation coefficients ≥0.85 or ≤ −0.8 were considered positive or negative relationships, respectively, and only statistically significant associations were visualized (Benjamini–Hochberg [BH]–adjusted *q* < 0.05). Nodes represent bacterial taxa, and node size reflects relative abundance based on mRNA read counts. Edge thickness indicates the magnitude of the correlation coefficient; edges denote positive or negative correlations according to the sign of the SparCC correlation coefficient. Network density represents the proportion of observed edges relative to all possible edges, the clustering coefficient reflects the degree of local interconnectedness among neighboring nodes, and network centralization indicates the extent to which connectivity is dominated by a limited number of highly connected taxa.

The taxonomic origin of each gene in the mRNA cluster was determined using functionally annotated data from the NCBI nr protein database. Taxa detected in both rc-rRNA and mRNA profiles were defined as VTiFs, and network analyses were conducted targeting only VTiF [3, 36]. Two main genera were present in the network before treatment in all groups: *Streptococcus* genus group, mainly consisting of *S. mitis, Streptococcus oralis, Streptococcus pneumoniae*, and the *Actinomyces* genus group, mainly consisting of *Actinomyces naeslundii, Actinomyces johnsonii, Actinomyces oris*, and others (Fig. 6a–d). The network structure of H1 consisted primarily of these groups. In the G1 network structure, we observed *C. gingivalis* and *Selenomonas* sp., in addition to taxa detected in the H1 network structure. Moreover, we observed an increased in both number of nodes constituting the G1 network structure and its network complexity compared with the H1 network structure. In the DP1 and RP1 network structures, more taxa were added to the taxa comprising the G1 network structure, such as the red complex and *Prevotella* genera; however, these main network structures in DP1 and RP1 still comprised *Streptococcus* and *Actinomyces* genera.

In the network structure of each group, the number of nodes post-treatment were more than that of the pre-treatment network except for the G group; however, the key species central to the pre-treatment network remained largely unchanged. The number of edges and the value of clustering coefficients decreased in the H and G groups after treatment. In contrast, these values were higher in the DP and RP groups after treatment. Network density and centralization values decreased after treatment in all groups. We observed negative correlations among the network structures of G1, DP2, RP1, and RP2. In the network structure of G1, *Selenomonas* sp., an anaerobic bacterium and *R. aeria*, an aerobic bacterium, were negatively correlated. Moreover, we found negative correlations between obligate anaerobic bacteria, such as *Treponema* and *Tannerella* genera, and obligate aerobic and facultative anaerobic bacteria, such as *Neisseria, Actinomyces*, and *Streptococcus* genera in the network structures of DP2, RP1, and RP2. In terms of the difference between DP1 and RP1 in the network, all values of the network structure were greater in the DP1 than RP1, with the number of edges and clustering coefficient being particularly elevated. Additionally, *P. gingivalis, T. denticola,* and *T. forsythia* had positive correlations with each other and formed the same cluster in the network structure of DP1. However, we observed no positive or negative correlations between *P. gingivalis* and different taxa in the network structure of RP1. Furthermore, we observed no positive or negative correlations between *T. denticola* and *T. forsythia*, and these taxa had few connections with other taxa. *T. forsythia* and *Fusobacterium* sp., which are known to cause periodontal disease, were negatively correlated with aerobic bacteria in the RP1 network, whereas DP1 network structure showed no negative correlation. The number of edges, network density, and centralization values were much lower in the RP2 network than in the DP2 network. In the network structure of DP2, the overly dense edge aggregation in the cluster comprising primarily of *Streptococcus* and *Actinomyces* genera still remained similar to that in the network structure of DP1. In contrast, dense edge aggregation in the network structure of RP2 was clearly reduced compared to that of RP1.

## Discussion

Periodontal treatment alters the bacterial community and that bacteriological features contribute not only to the progression of periodontitis but also to differences in treatment efficacy. Our findings show differences in healing outcomes between DP and RP groups even without differences in PPD, BOP, smoking history, and RBL at baseline. These variations in healing may be partially explained by differences in the bacterial network structure at baseline, despite the similarities in the bacterial composition.

In the network analysis, bacterial groups centered on *Streptococcus* and *Actinomyces* genera were observed in all groups. As the progression moved from healthy sites to gingivitis sites, and from gingivitis to periodontitis sites, densely connected edges tended to form within these bacterial groups, and network centralization increased. Additionally, disease-associated bacteria, such as *C. gingivalis* and *Selenomonas* genus at gingivitis sites and the red complex at periodontitis sites, were added to the network. Therefore, the network structure of each group exhibited a transitional arrangement, supporting similar observations from the PCA. Together with the PCA/PERMANOVA results, the correlation networks provide a complementary view by highlighting taxon–taxon association patterns that are not captured by community-level ordination alone.

In the diversity analysis, we observed an increasing trend in α diversity from pre- to post-treatment in all groups, whereas a study that sequenced the 16S rRNA gene library prepared from bacterial DNA indicated a decrease in α diversity at periodontitis sites after treatment [14, 44, 45]. This discrepancy appears to arise from the presence of dead or inactive microbes in DNA-based studies. Indeed, the discrepancies between DNA- and RNA-based bacterial analysis results, as reported in other studies [46], suggest that the findings of the present study do not contradict those of the previous studies. Based on our RNA-based results, the richness of active bacterial species and their transcriptional activity after treatment were possibly elevated. In the β diversity analysis based on bacterial and functional data, differences in bacterial composition observed between pre-treatment groups were no longer apparent post-treatment, suggesting that both bacterial and functional gene compositions at diseased sites became more similar to those in a healthy state after periodontal treatment.

In bacterial networks of DP1 and RP1, although pathogenic bacteria including the red complex were present, their correlations were not dense. Instead, the emergence of the red complex disrupts homeostasis by causing health-associated bacteria such as those of the *Streptococcus* and *Actinomyces* genera [47] to become excessively active and influential. Health-associated bacteria are also present at periodontitis sites [48–50]. Although whether excessive interactions within these commensal bacterial networks cause or result from periodontitis remains unclear, these findings represent a “disruption” not observed in clinically healthy states such as H2, G2, or RP2, providing a new perspective on periodontitis as a dysbiosis-related disease. In H2, G2, and RP2, the formation of densely connected edges within these bacterial groups, including *Streptococcus* and *Actinomyces* genera, was alleviated. This was accompanied by reduced network density and centralization, and edges previously concentrated around these bacterial taxa became distributed among multiple other taxa. These findings suggest that such a stabilized network structure may indicate a healthy bacterial network state. Duran-Pinedo *et al*. [51] found that the number of hubs increased in stable periodontitis sites after treatment, enhancing resilience against future relapse, which aligns with our findings. In both DP and RP, negatively correlated edges increased after treatment. This is thought to result from the resolution of edge localization symbolized by clusters of *Streptococcus* and *Actinomyces* genera due to treatment, allowing these bacteria to antagonize periodontal pathogens.

Non-surgical treatment increased the DAT of aerobic and health-related bacteria, such as *Neisseria* [47] and decreased the DAT of anaerobic and periodontal pathogens including the red complex in each group, consistent with previous reports [14, 51, 52]. By contrast, *P. gingivalis* increased at H2 sites in individuals with DP2 sites, which may partly reflect residual disease not only at anterior sites but also in the posterior region. We observed no statistical differences in β diversity between DP2 and RP2; however, we observed differences in DAT and network structure. These differences may contribute to the persistence of periodontitis. For example, bacteria taxa such as *Bacteroidetes* [G-5] sp. and *P. endodontalis* were more abundant in DP2 than in RP2. These bacterial taxa were also more abundant in P1 than in G1 and are known to be highly prevalent in patients with periodontitis [53, 54]. Additionally, *P. endodontalis* plays a crucial role in the onset and progression of periodontitis by acquiring iron and evading immune responses [55]. Therefore, these bacteria are suspected to contribute to periodontitis onset and interfere with its treatment. In the cross-sectional comparison of DP1 and RP1, the analyses of α and β diversity, bacterial composition, and functional gene expression did not reveal significant differences, making it difficult to identify bacteria associated with poor prognosis. However, using the longitudinal design employed in this study, we successfully highlighted bacterial taxa that may facilitate or impede the resolution of periodontitis.

After treatment, *N. elongata* and *Capnocytophaga sputigena* were identified as DAT, increased in the RP group but not in the DP group. Notably, these two bacterial taxa were absent from the DP2 network, while in the RP2 network, they exhibited negative correlations and antagonistic interactions with anaerobic groups such as *Prevotella oralis* and *Prevotella dentalis*. Similarly, *R. aeria* was significantly more abundant at RP2 than at DP2. This bacterial taxon was present in the networks of DP1, DP2, RP1, and RP2; however, only RP2 was negatively correlated with anaerobic bacteria. Pozhitkov *et al*. [56] reported that in healthy oral conditions included contributions from several bacteria, such as *N. elongata* [57]. *Rothia* and *Neisseria* have an inverse relationship with inflammatory cytokines, and their growth in the presence of nitrates may alleviate inflammation [58]. These findings suggest that these bacteria contribute to the resolution of periodontitis by exhibiting antagonistic interactions with anaerobic bacteria in the post-treatment environment. Building on this concept, our findings suggest that bacterial network architecture itself may be therapeutically relevant. In addition to targeting individual taxa, approaches that reconstruct network structures—such as prebiotic-like or bacteriophage-based strategies—may facilitate favorable healing-associated communities.

The downregulation of genes involved in polysaccharide biosynthesis suggests evasion of the host immune system through polysaccharide biosynthesis [59], which was observed in both the H and G groups after treatment. That expression of genes encoding polysaccharide biosynthesis proteins also decreased in the H group indicates that even at H sites, treatment reduced pathogenicity, suggesting that the treatment was effective. DEGs common to the DP and RP groups before and after treatment included genes encoding glycine dehydrogenase (decarboxylating) β subunit and cleaved adhesin domain protein. These genes decreased following treatment in the RP group but increased in the DP group. These genes encoding proteins are related to amino acid metabolism by glycine degradation and increased infectivity by adhesion to the tooth surface and host cells, respectively [60, 61], which suggests that the functional genes may inhibit the healing of periodontitis. In the comparison between the DP and RP groups, genes encoding the C-terminal domain protein, type VI secretion protein, and endopeptidase were detected as DEGs that were upregulated in DP2 compared with RP2, identifying them as characteristic genes of unhealed periodontitis sites after treatment. C-terminal domain is considered to contribute to the virulence of *P. gingivalis*, as found in its genes including gingipains and peptidyl deaminases [62]. The type VI secretion system is a gene cluster of gram-negative bacteria that encodes a protein complex responsible for injecting effector molecules into other bacteria and eukaryotes [63]. Endopeptidases, including gingipain and collagenase, are involved in the destruction of periodontal tissues [64, 65]. Based on these findings and previous reports, these genes are suggested to inhibit the healing of periodontitis. Targeting these genes may provide a potential strategy to promote healing in periodontitis.

In the H sites, where no clinical changes were observed between H1 and H2, the increase in α diversity, changes in β diversity of functional genes, the presence of DAT and DEGs, and the resolution of network centralization indicate that the bacterial community changed following treatment. Huang *et al*. [15] demonstrated that changes in the microbiome occur before the onset of gingival inflammation during the pre-phase of experimental gingivitis, which indicates a variation in the composition of the bacterial community even within a healthy state. In the RP2 network, even within the same healthy state, anaerobic bacterial species that were not present within H2 or G2 of the network were observed. Through RNA-level analysis we succeeded in observing such distinct differences, highlighting the functional changes in the bacterial community. These findings suggest that even if periodontitis is resolved and, due to the disappearance of β diversity differences and the increase in health-associated bacteria, the condition appears healthy, the bacterial network structure differs from that of the originally healthy sites. This may explain one of the reasons why teeth with a history of periodontitis are more prone to recurrence. The fact that the network structure of RP2 differs from that of H2 and G2 can serve as one piece of evidence supporting the importance of continuous maintenance.

One limitation of this study is that post-treatment sampling was conducted at least three months after non-surgical periodontal therapy, with some variability in follow-up timing across individuals. Although major clinical and microbiological changes are known to occur within the early post-treatment phase [66, 67], we cannot completely exclude the possibility that subtle time-dependent bacterial shifts may have influenced the observed results. Because all patients underwent full-mouth non-surgical periodontal therapy, recolonization from posterior sites was likely minimized; however, residual molar pockets remained in some patients. This site-specific design is therefore a limitation, and studies including posterior sites are warranted. Additionally, the findings were based solely on RNA analysis and the functional annotation framework used in this study, and a more comprehensive assessment of the bacterial community may be achieved using updated annotation databases and a multi-omics approach [68]. Furthermore, dual sequencing approaches that include host response analysis should be considered because periodontitis is fundamentally driven by host immune reactions [51].

## Supplementary Material

Supplementary_material_ycag092

## Data Availability

The datasets generated for this study can be found in the DNA Data Bank of Japan (DDBJ) under the accession number for RNA sequencing PRJDB18491.
