# Supplementary material for: Metatranscriptomic insights into microbial network modulation and pathogen dynamics underlying healing outcomes in non-surgical periodontal treatment
Source: ISME Commun. 2026 Apr 11;6(1):ycag092. doi: 10.1093/ismeco/ycag092 (PMC13235731; doi:10.1093/ismeco/ycag092)
Supplement: Supplementary_material_ycag092 [file supplementary_material_ycag092.zip › Text_S1_ycag092.docx]

**Supplementary information – Text S1**

**Content of Text S1**

1. **SUPPLEMENTAL MATERIAL AND METHODS**
2. **REFERENCES**

**1. SUPPLEMENTAL MATERIALS AND METHODS**

**RNA extraction**

RNA was extracted using the PowerMicrobiome RNA Isolation Kit (Qiagen, Venlo, Netherlands). Purified RNA was quantified using a Quantus fluorometer (Promega, Madison, WI, USA), and RNA quality was evaluated using an Agilent 2100 bioanalyzer system (Agilent Technologies, Santa Clara, CA, USA) as described in previous studies(1-3).

**Complementary DNA synthesis, library preparation, and Illumina sequencing**

Purified RNA was polyadenylated using an A-Plus Poly(A) Polymerase Tailing Kit (Epicentre Biotechnologies, Madison, WI, USA) and concentrated by ethanol precipitation using the Dr. GenTLE Precipitation Carrier (Takara Bio, Shiga, Japan). The polyadenylated RNA was reverse-transcribed into complementary DNA (cDNA) using the SMART-Seq v4 Ultra® Low Input RNA Kit for Sequencing (Takara)(3). Metatranscriptome sequencing libraries were constructed using a Nextera XT DNA Library Preparation Kit (Illumina, San Diego, CA, USA). The amplified cDNA was quantified by performing real-time polymerase chain reaction on a LightCycler (Roche Diagnostics, Mannheim, Germany) using a KAPA Library Quantification Kit (KAPA Biosystems, Wilmington, MA, USA). Library quality was evaluated using the Agilent 2100 Bioanalyzer. The prepared samples were pooled, and the Illumina MiSeq system platform was used to generate 300-base pair (bp) paired-end reads.

**Processing and analyzing Illumina sequencing data**

The sequencing data obtained in this study were analyzed together with data downloaded from the DNA Data Bank of Japan (DDBJ) (DRA011737), which were derived from 21 patients before non-surgical treatment in a previous study. The Illumina sequencing data were processed and analyzed as described previously(1, 2). Raw reads were trimmed using the Trimmomatic software version 0.32(4). Sequences derived from the host were removed using DeconSeq software, version 0.4.3(5). The trimmed data were separated into paired and unpaired reads using cmpfastq software(6). Paired reads were then merged using fastq-join(7) to facilitate downstream taxonomic and functional analysis.

16S rRNA analysis and OTUs identification were conducted using EMIRGE pipeline with only paired reads(8). Reconstructed 16S rRNA genes (rc-rRNA) are nearly full-length sequences assembled from paired-end reads. The number of reads for each rc-rRNA was calculated as the abundance value of its corresponding 16S rRNA OTU. We classified the representative sequence of each rc-rRNA OTU as similar to that in HOMD, version 13.2(9), using the Basic Local Alignment Search Tool N (BLASTN)(10, 11). The number of OTUs and Shannon index were used to estimate the α diversity indices. Rarefaction curves were drawn from abundance values calculated from the number of OTUs and species aligned to HOMD utilizing the rarefaction single command in Mothur software, version 1.48.0(12). The abundance of all rc-rRNA OTUs was normalized using centered log-ratio (CLR) values(13). Subsequently, community diversity was compared using PERMANOVA and visualized using PCA, both based on the Aitchison distance. In addition, all reads, including non-merged and unpaired reads, were formed into OTUs using Cluster Database at High Identity with Tolerance software. The 16S rRNA OTUs were removed by similarity comparison using BLASTN against SILVA (release 119)(14). The remaining OTUs were assumed to be derived from mRNA. Based on previous studies(1, 2), the mRNA OTUs were used to identify putative virulence factors using Basic Local Alignment Search Tool X (BLASTX)(15) against the NCBI nr protein database (as of October 31, 2014), VFDB (as of February 9, 2015), and MvirDB (as of October 9, 2014), and protein function profiles were obtained.

The abundance values of all mRNA OTUs were normalized by conversion to transcripts per million (TPM) to account for differences in gene length and library size. Only the mRNA OTUs with a prevalence of at least 50% in at least one group were included in the analysis. Additionally, batch effects were recognized between the data from previous studies and those used in this study(16); therefore, the Conditional Quantile Regression (ConQuR) approach was employed to remove these batch effects.(17) The Bray–Curtis distance was used for PERMANOVA and PCoA. The Bray–Curtis dissimilarity of each group was calculated using the R package vegan (version 4.2.1; R Foundation for Statistical Computing, Vienna, Austria). We used the R packages tidyverse(18) and ggplot2(19) to generate the PCoA plots. Additionally, multi-group analysis of differentially abundant taxa from rc-rRNA and differentially expressed genes from mRNA was conducted using the R package ANCOM-BC2(20).

Only the taxa identified in both 16S rRNA and mRNA OTUs were included for further analyses, and these taxa were defined as viable taxa with *in situ* functions (VTiFs)(1, 2). To understand the detailed positive and negative correlation relationships in the mRNA profiles of VTiF, we extracted only the VTiFs present in at least 50% of the participants in at least one group, and used them for the creation of network structures. Correlation coefficients were calculated using the Sparse Correlations for Compositional data (SparCC) software(21) based on mRNA taxonomic abundances. Taxon pairs with SparCC values of ≥ 0.85 and ≤ −0.8 were regarded as positive and negative relationships, respectively. Only taxon pairs with significance identified using the Benjamini–Hochberg (BH) method (*q* < 0.05) were visualized using Cytoscape software, version 3.10.2(22). The number of nodes and edges and the values of network density, clustering coefficient, and network centralization were also calculated for each group using Cytoscape.

**2. REFERENCES**

1. Funahashi K, Shiba T, Watanabe T, Muramoto K, Takeuchi Y, Ogawa T, Izumi Y, Sekizaki T, Nakagawa I, Moriyama K. 2019. Functional dysbiosis within dental plaque microbiota in cleft lip and palate patients. Progress in Orthodontics 20.

2. Shiba T, Watanabe T, Kachi H, Koyanagi T, Maruyama N, Murase K, Takeuchi Y, Maruyama F, Izumi Y, Nakagawa I. 2016. Distinct interacting core taxa in co-occurrence networks enable discrimination of polymicrobial oral diseases with similar symptoms. Sci Rep 6:30997.

3. Nemoto T, Shiba T, Komatsu K, Watanabe T, Shimogishi M, Shibasaki M, Koyanagi T, Nagai T, Katagiri S, Takeuchi Y, Iwata T. 2021. Discrimination of Bacterial Community Structures among Healthy, Gingivitis, and Periodontitis Statuses through Integrated Metatranscriptomic and Network Analyses. Msystems 6.

4. Bolger AM, Lohse M, Usadel B. 2014. Trimmomatic: a flexible trimmer for Illumina sequence data. Bioinformatics 30:2114-20.

5. Schmieder R, Edwards R. 2011. Fast identification and removal of sequence contamination from genomic and metagenomic datasets. PLoS One 6:e17288.

6. Shen FF, Long Y, Li FY, Ge GD, Song GL, Li Q, Qiao ZG, Cui ZB. 2020. *De novo* transcriptome assembly and sex-biased gene expression in the gonads of Amur catfish (Silurus asotus). Genomics 112:2603-2614.

7. Aronesty E. 2013. Comparison of Sequencing Utility Programs. Open Bioinforma J 7:1-8.

8. Miller CS, Baker BJ, Thomas BC, Singer SW, Banfield JF. 2011. EMIRGE: reconstruction of full-length ribosomal genes from microbial community short read sequencing data. Genome Biology 12.

9. Chen T, Yu WH, Izard J, Baranova OV, Lakshmanan A, Dewhirst FE. 2010. The Human Oral Microbiome Database: a web accessible resource for investigating oral microbe taxonomic and genomic information. Database-the Journal of Biological Databases and Curation doi:ARTN baq01310.1093/database/baq013.

10. Altschul SF, Gish W, Miller W, Myers EW, Lipman DJ. 1990. Basic local alignment search tool. J Mol Biol 215:403-10.

11. Camacho C, Coulouris G, Avagyan V, Ma N, Papadopoulos J, Bealer K, Madden TL. 2009. BLAST+: architecture and applications. BMC Bioinformatics 10:421.

12. Schloss PD, Westcott SL, Ryabin T, Hall JR, Hartmann M, Hollister EB, Lesniewski RA, Oakley BB, Parks DH, Robinson CJ, Sahl JW, Stres B, Thallinger GG, Van Horn DJ, Weber CF. 2009. Introducing mothur: Open-Source, Platform-Independent, Community-Supported Software for Describing and Comparing Microbial Communities. Applied and Environmental Microbiology 75:7537-7541.

13. Gloor GB, Macklaim JM, Pawlowsky-Glahn V, Egozcue JJ. 2017. Microbiome Datasets Are Compositional: And This Is Not Optional. Front Microbiol 8:2224.

14. Shi YM, Tyson GW, DeLong EF. 2009. Metatranscriptomics reveals unique microbial small RNAs in the ocean's water column. Nature 459:266-U154.

15. Gish W, States DJ. 1993. Identification of Protein Coding Regions by Database Similarity Search. Nature Genetics 3:266-272.

16. Regueira-Iglesias A, Balsa-Castro C, Blanco-Pintos T, Tomás I. 2023. Critical review of 16S rRNA gene sequencing workflow in microbiome studies: From primer selection to advanced data analysis. Molecular Oral Microbiology 38:347-399.

17. Ling WD, Lu JY, Zhao N, Lulla A, Plantinga AM, Fu WJ, Zhang A, Liu HJ, Song H, Li ZG, Chen J, Randolph T, Koay WL, White JR, Launer LJ, Fodor AA, Meyer KA, Wu MC. 2022. Batch effects removal for microbiome data via conditional quantile regression. Nature Communications 13.

18. Wickham H AM, Bryan J, Chang W, McGowan L, François R, Grolemund G, Hayes A, Henry L, Hester J, Kuhn M, Pedersen T, Miller E, Bache S, Müller K, Ooms J, Robinson D, Seidel D, Spinu V, Takahashi K, Vaughan D, Wilke C, Woo K, Yutani H. 2019. Welcome to the Tidyverse. Journal of Open Source Software 4:43.

19. Wickham H. 2016. Programming with ggplot2, p 241-253, ggplot2: Elegant Graphics for Data Analysis doi:10.1007/978-3-319-24277-4_12. Springer International Publishing, Cham.

20. Lin H, Peddada SD. 2023. Multigroup analysis of compositions of microbiomes with covariate adjustments and repeated measures. Nat Methods doi:10.1038/s41592-023-02092-7.

21. Friedman J, Alm EJ. 2012. Inferring Correlation Networks from Genomic Survey Data. Plos Computational Biology 8.

22. Smoot ME, Ono K, Ruscheinski J, Wang PL, Ideker T. 2011. Cytoscape 2.8: new features for data integration and network visualization. Bioinformatics 27:431-432.
