# Supplementary material for: Metatranscriptomic insights into microbial network modulation and pathogen dynamics underlying healing outcomes in non-surgical periodontal treatment
Source: ISME Commun. 2026 Apr 11;6(1):ycag092. doi: 10.1093/ismeco/ycag092 (PMC13235731; doi:10.1093/ismeco/ycag092)
Supplement: Supplementary_material_ycag092 [file supplementary_material_ycag092.zip › Figure_S1_to_S13_revised2_ycag092.pdf]

Figure S1

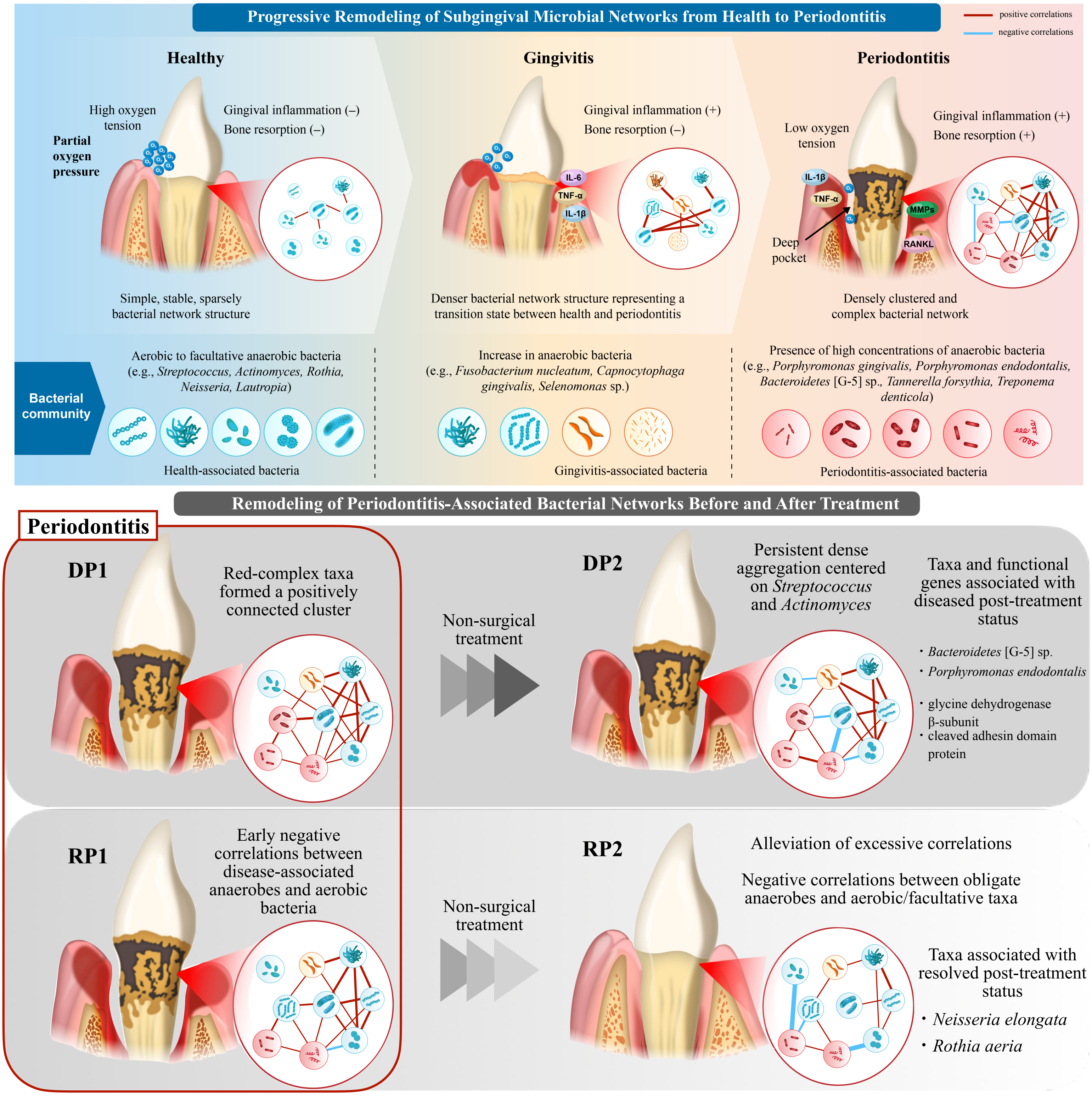

Figure S1 Conceptual summary of bacterial ecological transitions across periodontal conditions and treatment outcome-dependent remodeling of periodontitis-associated networks. (a) Conceptual illustration of ecological changes across healthy, gingivitis, and periodontitis sites. This panel illustrates shifts in bacterial community composition, oxygen tension, inflammatory status, and bacterial network structure. Healthy sites are characterized by dominance of aerobic to facultative anaerobic bacteria, relatively high oxygen levels, minimal inflammation, and a simple, sparsely connected network. Gingivitis represents a transitional state with reduced oxygen availability, increasing anaerobic taxa, early inflammation, and a denser network structure. Periodontitis is characterized by deep periodontal pockets, pronounced inflammation, a highly anaerobic environment, dominance of anaerobic pathogens, and a markedly dense and complex bacterial network. This panel summarizes the pre-treatment ecological continuum of periodontal disease. (b) Conceptual illustration of the principal findings of this study regarding outcome-dependent remodeling of periodontitis-associated bacterial networks before and after non-surgical periodontal treatment. Before treatment, both diseased periodontitis (DP1) and resolved periodontitis (RP1) sites showed excessive associations centered on *Streptococcus* and *Actinomyces*. In DP1, red-complex taxa (*Porphyromonas gingivalis*, *Tannerella forsythia*, and *Treponema denticola*) formed a positively connected cluster, whereas RP1 showed early negative correlations between disease-associated anaerobes and aerobic bacteria. After treatment, the overly dense aggregation centered on *Streptococcus* and *Actinomyces* persisted in diseased sites (DP2), whereas excessive correlations were alleviated in resolved sites (RP2). Negative correlations between obligate anaerobes and aerobic/facultative taxa were observed in RP2. In addition, *Bacteroidetes* [G-5] sp., *Porphyromonas endodontalis*, glycine dehydrogenase β-subunit, and cleaved adhesin domain protein are shown as features associated with diseased post-treatment status, whereas *Neisseria elongata* and *Rothia aeria* are shown as taxa associated with resolved post-treatment status. DP and RP indicate diseased periodontitis and resolved periodontitis, respectively. The numbers “1” and “2” denote pre-treatment and post-treatment status, respectively.

Figure S2

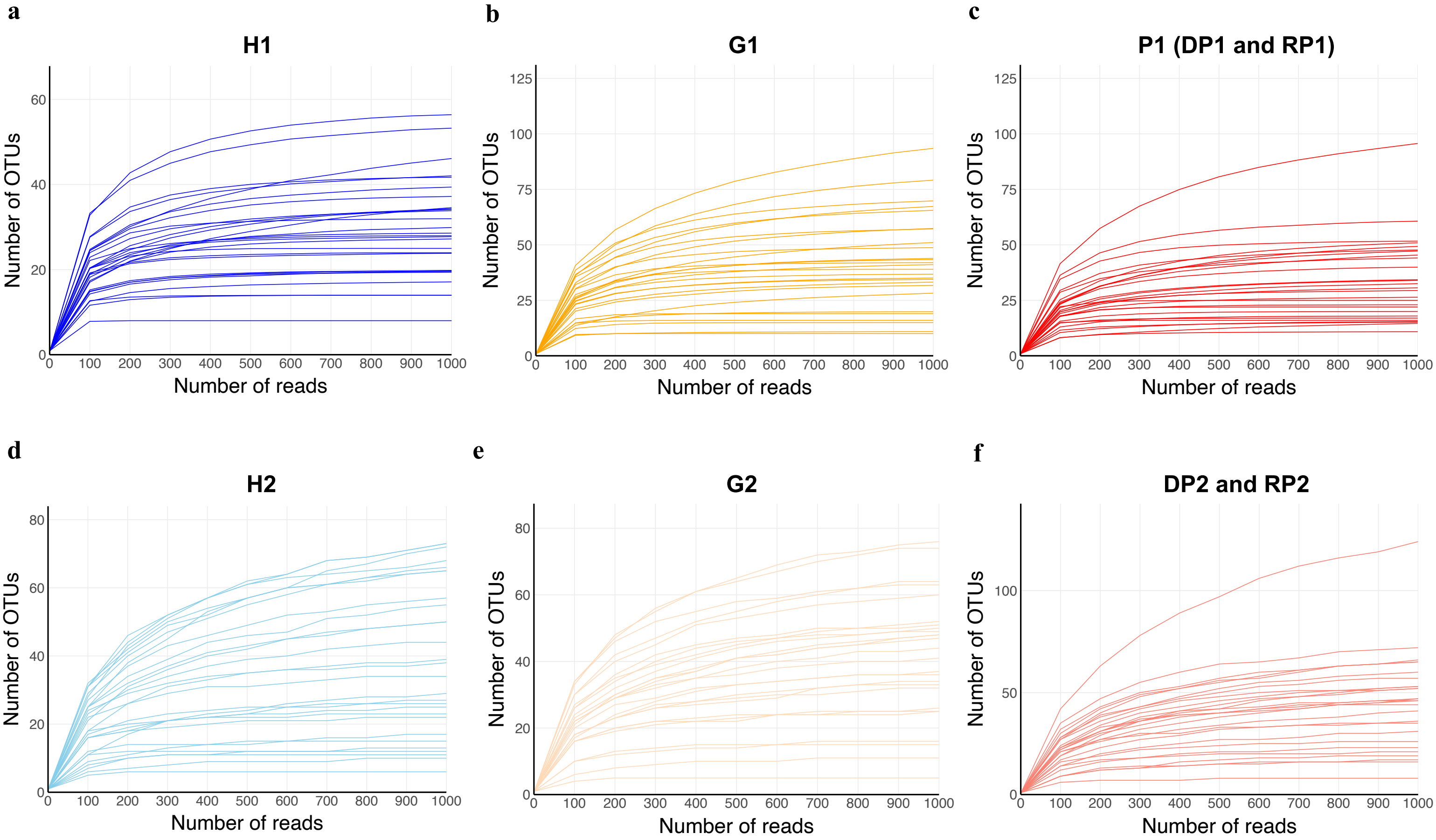

Figure S2 Rarefaction curves of pre- and post-treatment bacterial samples. Rarefaction curves show the number of observed operational taxonomic units (OTUs) according to sequencing depth. (a–c) Pre-treatment samples (H1, G1, and P1) from subjects with sufficient read counts are shown. (d–f) Post-treatment samples (H2, G2, DP2, and RP2) from subjects with sufficient read counts are shown. H, G, DP, and RP indicate healthy, gingivitis, diseased periodontitis, and resolved periodontitis sites, respectively. P1 indicates all pre-treatment periodontitis sites before classification into DP2 and RP2 outcome groups. The numbers "1" and "2" denote pre-treatment and post-treatment status, respectively.

Figure S3

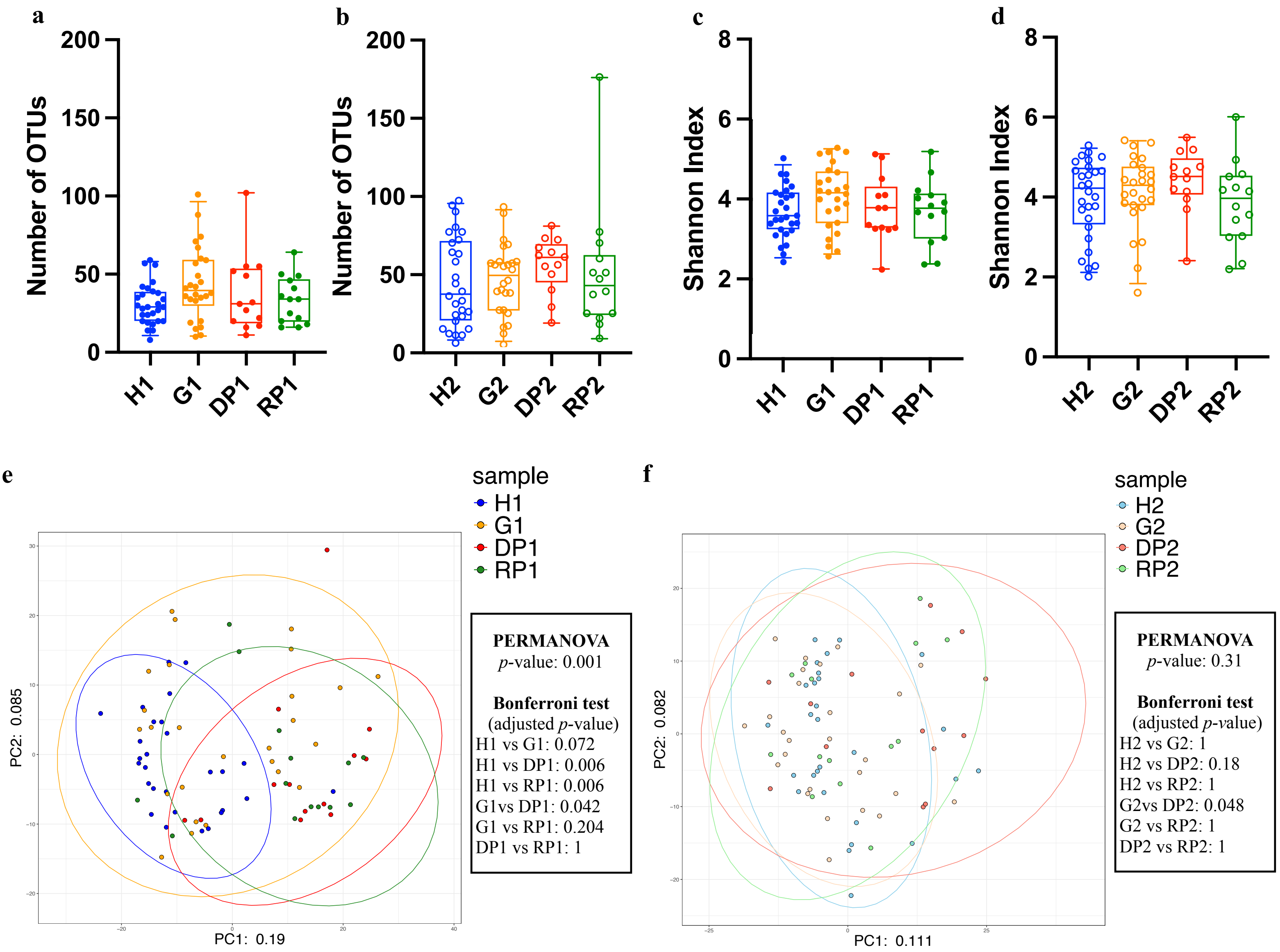

Figure S3 Cross-sectional comparisons of α and β diversity among periodontal condition groups before and after non-surgical treatment. (a–d) Alpha diversity was assessed by the number of operational taxonomic units (OTUs) (a, c) and the Shannon index (b, d) among the four groups at pre-treatment (H1, G1, DP1, RP1) and post-treatment (H2, G2, DP2, RP2), respectively. H, G, DP, and RP indicate healthy, gingivitis, diseased periodontitis, and resolved periodontitis sites, respectively. The numbers “1” and “2” denote pre-treatment and post-treatment status, respectively. Sample sizes were as follows; H: n = 28 (H1/H2), G: n = 26 (G1/G2), DP: n = 13 (DP1/DP2), and RP: n = 14 (RP1/RP2). Boxplots show the interquartile range (25th–75th percentiles) with the median as the center line; whiskers indicate the 95% confidence interval. Cross-sectional multi-group comparisons were assessed using the Kruskal–Wallis test followed by Dunn’s multiple-comparison test. No significant differences were detected among groups ($p \geq 0.05$).
(e, f) Principal component analysis based on Aitchison distance calculated from centered log-ratio –transformed rc-rRNA OTU abundances, are shown for pre-treatment (e) and post-treatment (f) samples. Each point represents one sample and ellipses indicate 95% confidence intervals. Group differences were evaluated using permutational multivariate analysis of variance (PERMANOVA), followed by post hoc pairwise comparisons with Bonferroni adjustment.

Figure S4

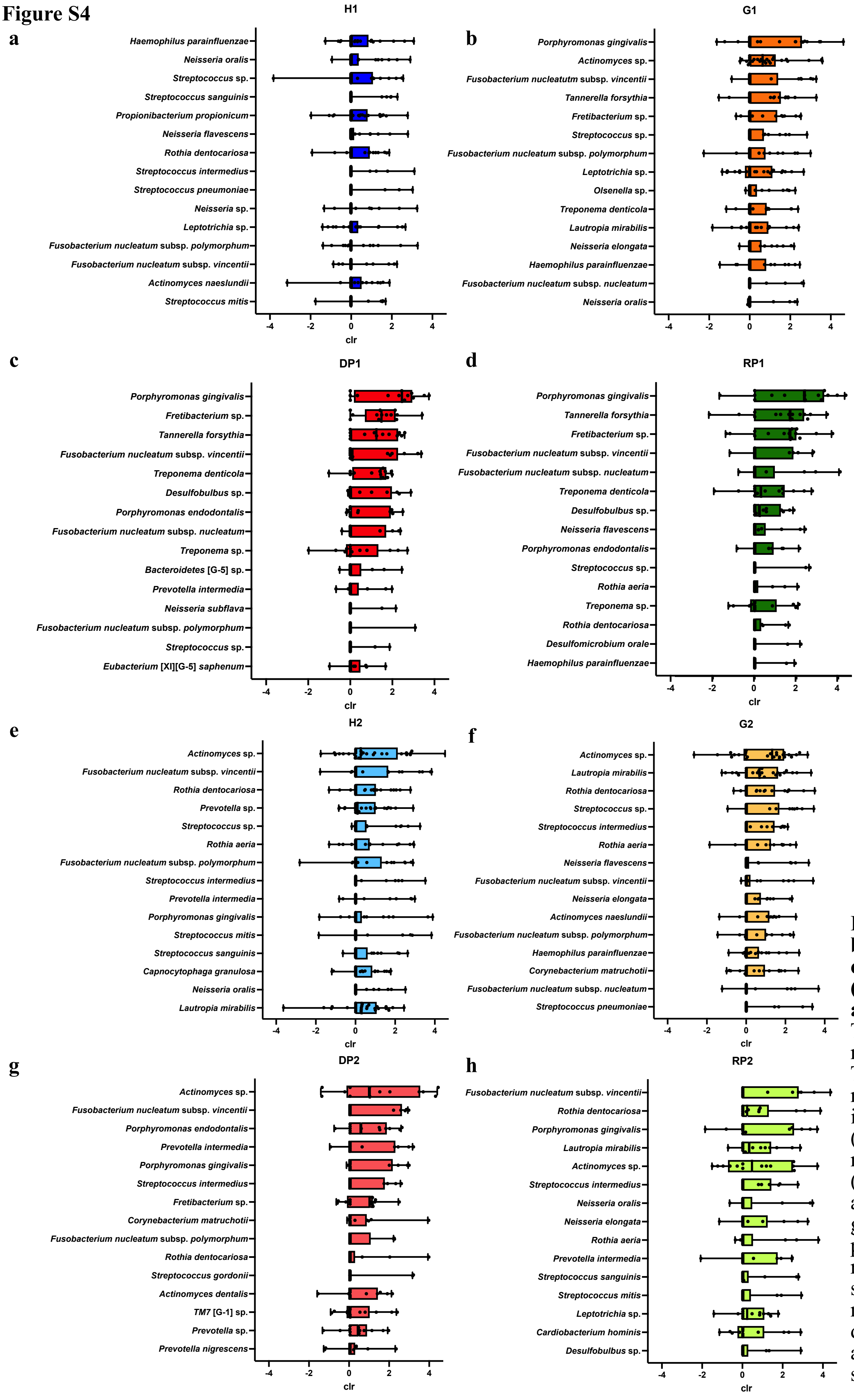

Figure S4 Top 15 bacterial taxa based on centered log-ratio (CLR)-transformed abundance values. Taxa are ordered by mean abundance values. The boxplots show the median (center line), interquartile range (box), and minimum and maximum values (whiskers). H, G, DP, and RP indicate healthy, gingivitis, diseased periodontitis, and resolved periodontitis sites, respectively. The numbers "1" and "2" denote pre-treatment and post-treatment status, respectively.

Figure S5

Taxon	H2/H1	G2/G1	DP2/DP1	RP2/RP1
Actinomyces dentalis	* 0.88	-0.16	0.62	0.72
Actinomyces gerencseriae	-0.13	0.08	* -1.05	1.38
Actinomyces israelii	0.01	-0.28	* -1.49	0.32
Actinomyces oris	* 1.86	* 0.79	0.00	0.47
Aggregatibacter sp.	0.34	* 0.97	0.00	0.00
Alloprevotella rava	0.00	0.00	* -1.50	0.00
Alloprevotella sp.	* 0.63 †	0.00	-2.72	-0.82
Alloprevotella tannerae	* 1.28	* -1.26	-0.58	0.17
Anaeroglobus geminatus	0.00	-0.47	* -2.11	0.58
Atopobium rimae	0.00	* -0.75	0.00	1.28
Bacteroidetes [G-5] sp.	* 1.41	-0.53	* -1.90	* -1.75
Campylobacter rectus	0.45	-0.43	* -0.95	-0.52
Capnocytophaga gingivalis	* -0.62	0.58	1.22	1.10
Capnocytophaga granulosa	0.05	0.10	* -2.00	-0.05
Capnocytophaga leadbetteri	* 0.64	0.28	-0.69	-0.80
Capnocytophaga sp.	0.13	-0.24	* -1.20	-1.21
Capnocytophaga sputigena	-0.07	0.16	-0.03	* 1.57
Catonella morbi	* 0.95 †	-0.33	0.20	0.04
Chloroflexi [G-1] sp.	0.00	* -0.70 †	* -1.41 †	0.77
Corynebacterium durum	-0.04	0.33	0.00	* 1.08 †
Corynebacterium matruchotii	0.06	* 1.12	-0.95	0.31
Desulfobulbus sp.	0.50	* -2.24 †	* -2.42	0.57
Desulfomicrobium orale	0.00	* -1.78	* -1.11	0.00
Eikenella corrodens	* -1.01	* -1.30	* -1.60	-0.31
Eubacterium [XI][G-5] *saphenum*	0.00	* -1.41	* -1.78	-0.97
Eubacterium [XI][G-6] *nodatum*	0.00	* -0.87	* -2.37	-0.61
Eubacterium [XI][G-7] *yurii*	* 0.83 †	* 0.69	0.09	0.00
Eubacterium [XIVa][G-1] *saburreum*	0.00	* 0.70	-1.13	-1.06
Filifactor alocis	0.00	* -1.56	* -1.81	0.51
Fretibacterium fastidiosum	-0.16	* -1.07	* -1.81	* -1.29
Fretibacterium sp.	0.31	* -1.84	* -2.06	-1.37
Fusobacterium nucleatun ss *vincentii*	* 0.80	-0.68	-0.49	* 1.36
Fusobacterium nucleatum ss *nucleatum*	* 0.85 †	-0.23	-1.06	1.62
Fusobacterium sp.	0.00	0.00	* 1.25	0.00
Gemella haemolysans	0.52	* 1.81 †	0.00	0.29
Gemella morbillorum	* 0.88	0.61	0.00	0.00
GN02 [G-1] sp.	0.00	* 0.86 †	0.00	0.00
Haemophilus parainfluenzae	-0.60	-0.29	* -1.28	* -1.53 †
Lachnoanaerobaculum umeaense	* 0.62 †	0.01	0.16	-0.27
Lachnospiraceae [G-8] sp.	0.00	0.00	* -0.82	-1.35
Leptotrichia buccalis	* 2.27	* -1.12	* -1.41	-1.61
Leptotrichia goodfellowii	* 1.07	0.34	0.00	0.93
Leptotrichia hofstadii	0.00	* -1.68	-2.05	1.02
Leptotrichia sp.	-0.32	-0.27	-0.23	* 1.43
Leptotrichia wadei	-0.03	* 0.62	* -1.91	0.00
Mogibacterium timidum	0.27	* -1.17	* -1.15	-0.42
Neisseria elongata	* -1.24	0.06	0.00	* 2.24
Neisseria flava	* 1.56	0.03	0.53	0.00
Neisseria flavescens	-0.11	* 0.76	0.00	-0.54
Neisseria sicca	0.00	* 1.11	0.00	0.00
Neisseria subflava	0.00	0.00	* -2.42	0.00
Olsenella sp.	-0.11	* -1.19	* -1.71	* 1.74
Oribacterium sp.	* 0.68	-0.28	0.00	0.00
Ottowia sp.	* -0.82	0.10	-0.69	-0.13
Parvimonas micra	0.00	* -0.81	* -1.61	0.50
Peptococcus sp.	* 0.84 †	-0.17	* -0.88	-0.21
Peptostreptococcaceae [XI][G-2] sp.	0.00	0.00	* -0.80	0.00
Peptostreptococcaceae [XI][G-4] sp.	0.00	* -1.36	* -1.26	-0.41
Porphyromonas catoniae	* 0.66	-0.04	0.00	0.00
Porphyromonas endodontalis	* 1.64 †	* -0.64	-0.58	-0.75
Porphyromonas gingivalis	* 1.28	* -1.19	* -1.66 †	-1.02
Porphyromonas sp.	-0.02	-0.09	* -2.24	-0.61
Prevotella intermedia	* 1.42	0.24	0.04	0.72
Prevotella melaninogenica	0.07	* -2.42	0.00	0.00
Prevotella nigrescens	0.21	-0.73	-0.51	* 1.12
Prevotella oris	* 0.78	-0.43	0.56	0.61
Prevotella sp.	* 1.00	0.03	-0.38	-0.53
Pseudoramibacter alactolyticus	0.00	* 0.75	* -1.72	-0.44
Rothia mucilaginosa	* 1.38	-0.06	-0.31	0.00
Selenomonas sp.	0.00	-0.38	* 1.92	-0.48
Solobacterium moorei	* 1.31	0.11	-0.33	0.90
Streptococcus anginosus	0.00	* 1.26	-0.41	0.00
Streptococcus intermedius	0.22	0.21	* 1.12	* 1.25
Streptococcus mitis	0.54	-0.56	-0.75	* 3.30
Streptococcus mutans	* 0.92	0.51	-1.00	0.00
Streptococcus pneumoniae	0.00	* 1.04	0.00	0.00
Streptococcus sanguinis	* -0.56	-0.40	0.00	0.63
Streptococcus sp.	0.36	0.60	-1.08	* -2.27
Tannerella forsythia	* -0.88	* -1.43	* -2.30	-0.97
Tannerella sp.	0.57	-0.48	-0.15	* -1.36
TM7 [G-1] sp.	* 0.77	-0.49	-0.65	-0.35
Treponema denticola	0.34	* -1.75	* -1.90	* -1.84 †
Treponema lecithinolyticum	0.00	0.00	* -2.07	-0.25
Treponema maltophilum	0.00	0.00	* -1.15	1.09

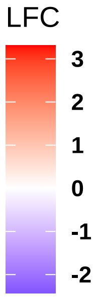

Figure S5 Comparative overview of differentially abundant taxa (DAT) detected across the four groups based on Human Oral Microbiome Database (HOMD) annotation. H, G, DP, and RP indicate healthy, gingivitis, diseased periodontitis, and resolved periodontitis sites, respectively. The numbers “1” and “2” denote pre-treatment and post-treatment status, respectively. Sample sizes were n = 28 for H, n = 26 for G, n = 13 for DP, and n = 14 for RP. This figure summarizes DAT identified by ANCOM-BC2 in longitudinal pre- and post-treatment comparisons within the H, G, DP, and RP groups (H2/H1, G2/G1, DP2/DP1, and RP2/RP1), displayed side by side to facilitate visual comparison of shared and group-specific abundance changes across groups. Taxa shown in the figure were selected based on statistical significance in at least one comparison (Benjamini–Hochberg-adjusted q < 0.05). For these displayed taxa, log fold change (LFC) values are shown for all four within-group comparisons, including comparisons that did not reach statistical significance. The color scale represents the LFC between post- and pre-treatment within each group. Asterisks (*) indicate statistically significant comparisons, and taxa that additionally passed the ANCOM-BC2 sensitivity analysis are indicated by a dagger symbol (†). Although all groups are displayed together for visual comparison, the statistical analyses shown in this figure are exclusively based on longitudinal (paired) comparisons within each group (H1 vs H2, G1 vs G2, DP1 vs DP2, and RP1 vs RP2). Abbreviations: DAT, differentially abundant taxon; LFC, log fold change; ANCOM-BC2, analysis of compositions of microbiomes with bias correction, version 2; HOMD, Human Oral Microbiome Database.

Figure S6

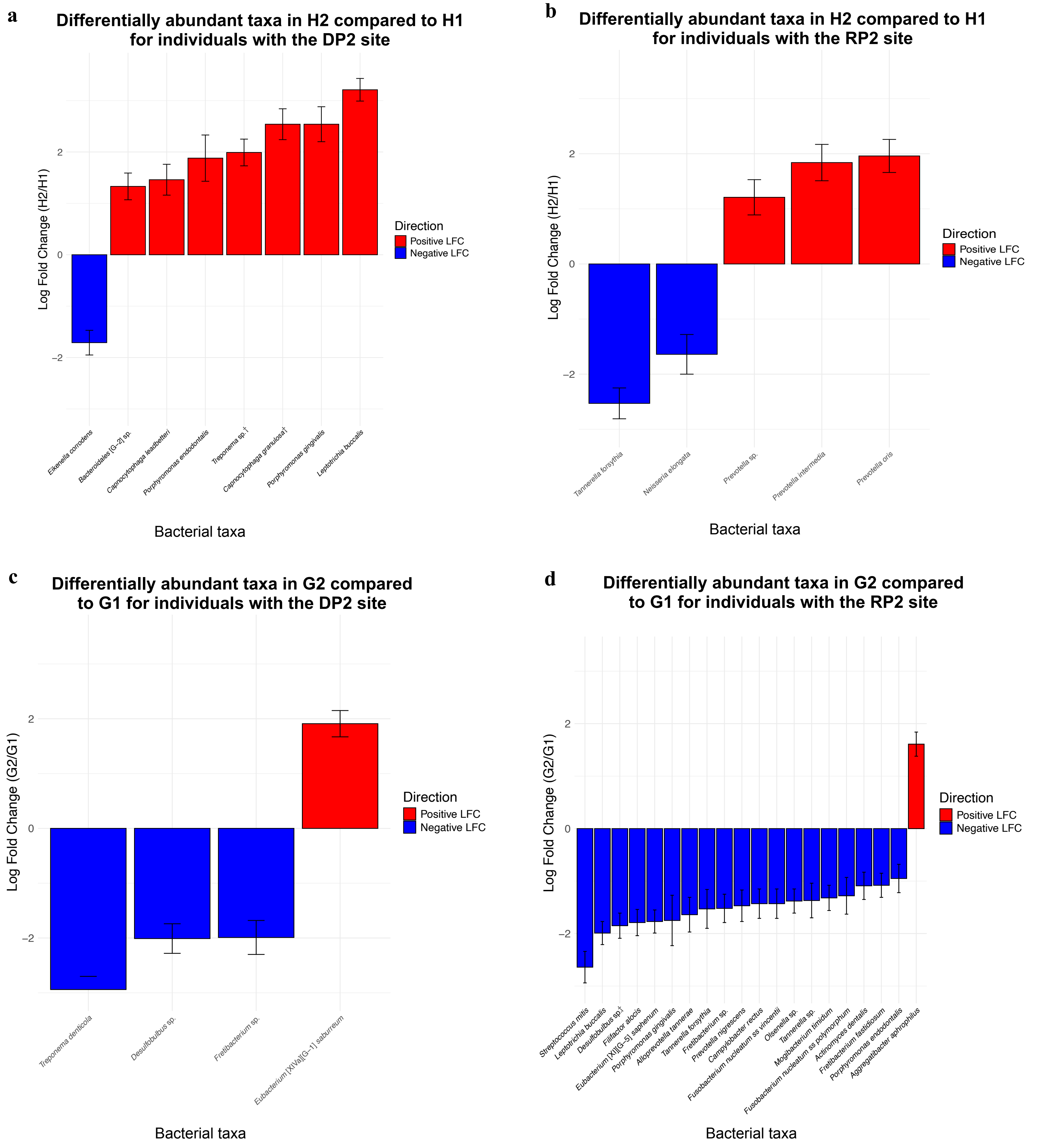

Figure S6 Differentially abundant taxa (DAT) identified in H sites from individuals who also had DP2 sites (a) or RP2 sites (b) within the same oral cavity, and in G sites from individuals who also had DP2 sites (c) or RP2 sites (d) within the same oral cavity. H, G, DP, and RP indicate healthy, gingivitis, diseased periodontitis, and resolved periodontitis sites, respectively. The numbers "1" and "2" denote pre-treatment and post-treatment status, respectively. Taxa with increased abundance are shown in red, whereas those with decreased abundance are shown in blue in each comparison. Bar height represents log fold change (LFC), and whiskers indicate ±1 standard error. Taxa that additionally passed the ANCOM-BC2 sensitivity analysis are marked with a dagger symbol (†). Abbreviations: DAT, differentially abundant taxon; LFC, log fold change; ANCOM-BC2, analysis of compositions of microbiomes with bias correction, version 2.

Figure S7

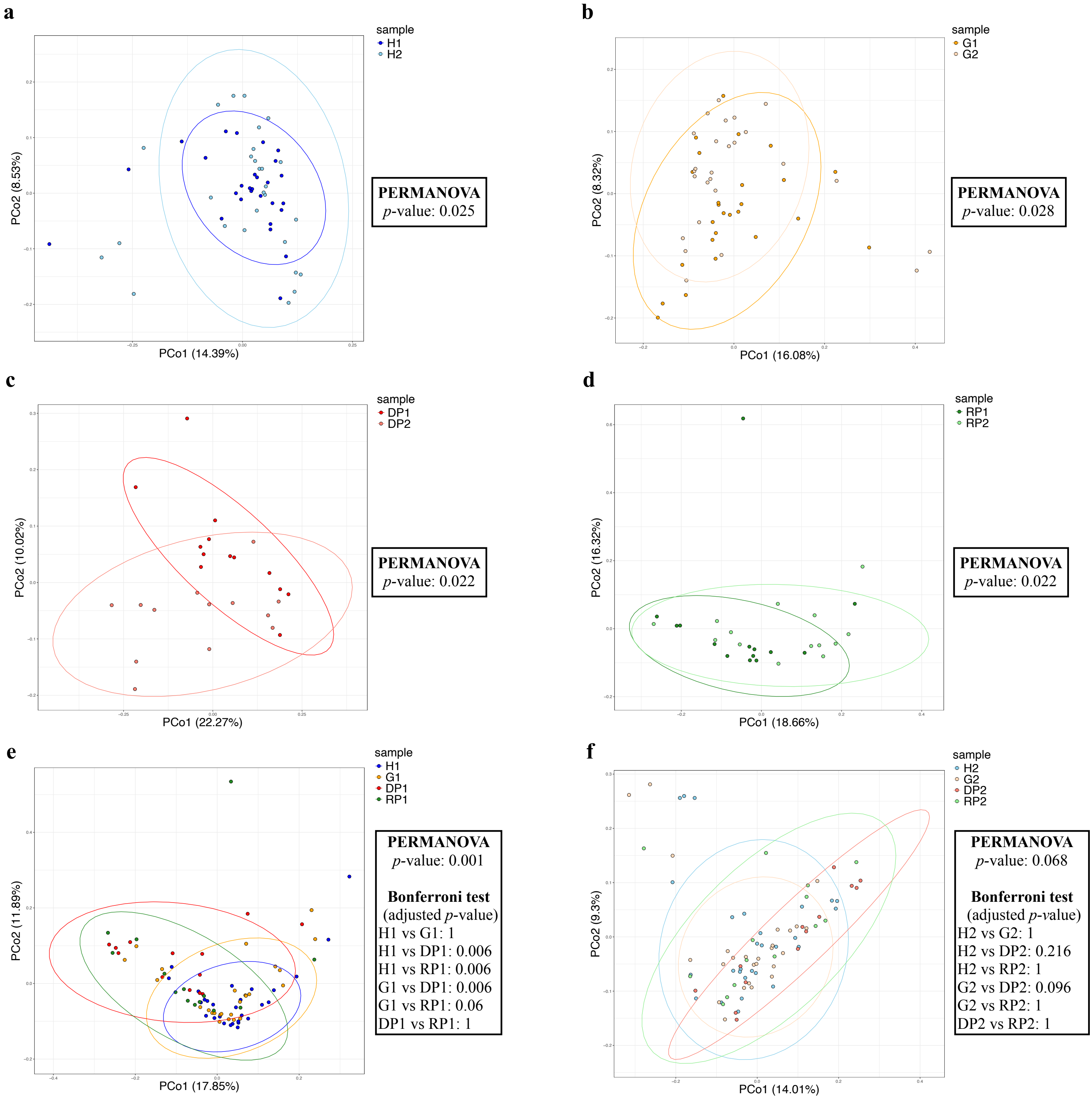

Figure S7 Principal coordinates analysis (PCoA) and permutational multivariate analysis of variance (PERMANOVA) based on Bray–Curtis distance calculated from functional gene abundance annotated against the National Center for Biotechnology Information non-redundant (NCBI nr) protein database. Darker colors indicate pre-treatment samples and lighter colors indicate post-treatment samples. Each point represents one sample, and ellipses indicate 95% confidence intervals. H, G, DP, and RP indicate healthy, gingivitis, diseased periodontitis, and resolved periodontitis sites, respectively. The numbers “1” and “2” denote pre-treatment and post-treatment status, respectively. Panels (a–d) show longitudinal comparisons between pre- and post-treatment samples within the H, G, DP, and RP groups, respectively. Panels (e) and (f) show cross-sectional multi-group comparisons at pre-treatment and post-treatment, respectively. Abbreviations: PCoA, principal coordinates analysis; PERMANOVA, permutational multivariate analysis of variance; NCBI nr, National Center for Biotechnology Information non-redundant protein database.

Figure S8

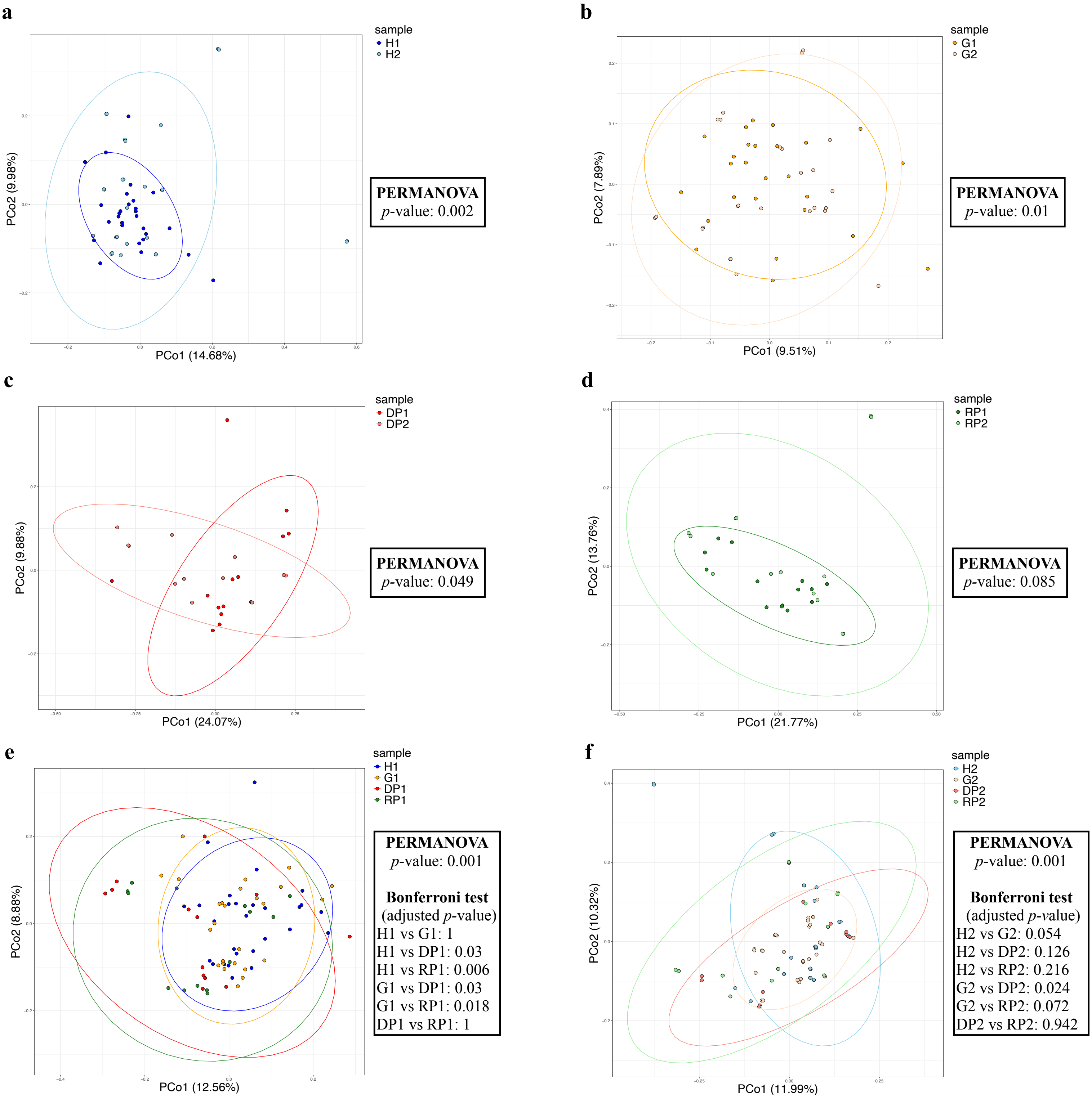

Figure S8 Principal coordinates analysis (PCoA) and permutational multivariate analysis of variance (PERMANOVA) based on Bray–Curtis distance calculated from functional gene abundance annotated against the Virulence Factors of Pathogenic Bacteria database (VFDB). Darker colors indicate pre-treatment samples and lighter colors indicate post-treatment samples. Each point represents one sample, and ellipses indicate 95% confidence intervals. H, G, DP, and RP indicate healthy, gingivitis, diseased periodontitis, and resolved periodontitis sites, respectively. The numbers “1” and “2” denote pre-treatment and post-treatment status, respectively. Panels (a–d) show longitudinal comparisons between pre- and post-treatment samples within the H, G, DP, and RP groups, respectively. Panels (e) and (f) show cross-sectional multi-group comparisons at pre-treatment and post-treatment, respectively. Abbreviations: PCoA, principal coordinates analysis; PERMANOVA, permutational multivariate analysis of variance; VFDB, Virulence Factors of Pathogenic Bacteria database.

Figure S9

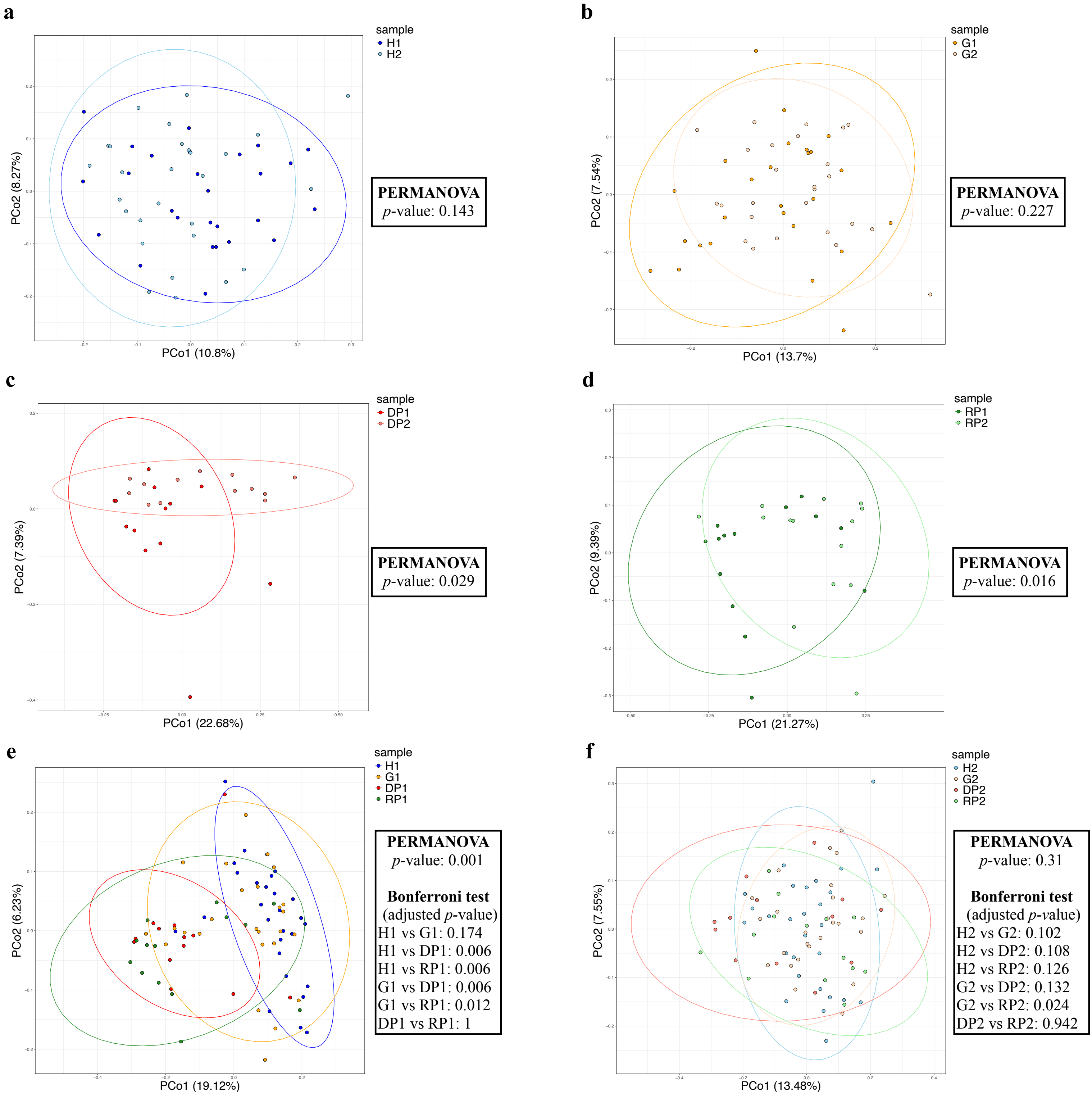

Figure S9 Principal coordinates analysis (PCoA) and permutational multivariate analysis of variance (PERMANOVA) based on Bray–Curtis distance calculated from functional gene abundance annotated against the Microbial Virulence Database (MvirDB). Darker colors indicate pre-treatment samples and lighter colors indicate post-treatment samples. Each point represents one sample, and ellipses indicate 95% confidence intervals. H, G, DP, and RP indicate healthy, gingivitis, diseased periodontitis, and resolved periodontitis sites, respectively. The numbers "1" and "2" denote pre-treatment and post-treatment status, respectively. Panels (a–d) show longitudinal comparisons between pre- and post-treatment samples within the H, G, DP, and RP groups, respectively. Panels (e) and (f) show cross-sectional multi-group comparisons at pre-treatment and post-treatment, respectively. Abbreviations: PCoA, principal coordinates analysis; PERMANOVA, permutational multivariate analysis of variance; MvirDB, Microbial Virulence Database.

Figure S10

a

	H1 vs H2	G1 vs G2	DP1 vs DP2	RP1 vs RP2
NCBI nr	0.025	0.028	0.022	0.022
VFDB	0.002	0.01	0.049	0.085
MvirDB	0.143	0.227	0.029	0.016

b

**Pre-treatment**	H1 vs G1	H1 vs DP1	H1 vs RP1	G1 vs DP1	G1 vs RP1	DP1 vs RP1
NCBI nr	1	0.006	0.006	0.006	0.06	1
VFDB	1	0.03	0.006	0.03	0.018	1
MvirDB	0.174	0.006	0.006	0.006	0.012	1
**Post-treatment**	H2 vs G2	H2 vs DP2	H2 vs RP2	G2 vs DP2	G2 vs RP2	DP2 vs RP2
NCBI nr	1	0.216	1	0.096	1	1
VFDB	0.054	0.126	0.216	0.024	0.072	0.534
MvirDB	0.102	0.108	0.126	0.132	0.024	0.942

Figure S10 Summary of PERMANOVA using Bray–Curtis distance based on functional gene abundance from each database. H, G, DP, and RP indicate healthy, gingivitis, diseased periodontitis, and resolved periodontitis sites, respectively. The numbers "1" and "2" denote pre-treatment and post-treatment status, respectively. Values with p or adjusted $p < 0.01$ are marked in dark orange, and those with $0.01 \leq p$ or adjusted $p < 0.05$ are marked in light orange. (a) PERMANOVA (p value) for longitudinal comparisons in each database. (b) Bonferroni-adjusted p values for cross-sectional comparisons in each database. Abbreviations: PERMANOVA, permutational multivariate analysis of variance.

Figure S11

Function	H2/H1	G2/G1	DP2/DP1	RP2/RP1
2-hydroxyglutaryl-CoA dehydratase	*0.2	0.2	0.3	*-1.5
3-dehydroquinate synthase	*0.5	* 0.4	0.0	0.0
30S ribosomal protein S16	*0.6	0.3	0.0	0.0
30S ribosomal protein S19	*0.3	-0.0	*0.7	0.0
4Fe-4S ferredoxin	0.0	* 0.6	0.0	-0.3
acetyl-CoA synthetase 2	0.0	* 0.5	0.4	*-1.4
acyl-CoA synthetase (AMP-forming)/AMP-acid ligase II	*-0.7†	* -0.8	*-1.1	0.7
AICARFT/IMPCHase bienzyme domain protein	*-0.6	* -0.5 †	*-0.4	*2.1†
alpha-2-macroglobulin family protein	0.0	* 0.6	0.2	-0.7
amino acid kinase family protein, partial	*-0.8†	* -1.1 †	-0.3	0.4
amino acid/peptide transporter	0.0	* 1.0 †	-0.1	-0.4
antibiotic resistance protein VanZ	*-0.5	* -0.4	*-1.0	*1.2
autotransporter adhesin, partial	0.0	* -0.6	*-1.3	*1.7
bifunctional protein FolD	*-0.5†	* -0.9	-0.1	*1.1
chaperonin GroL	0.0	* 1.1 †	0.0	0.0
chemotaxis protein CheC	0.0	* 0.4 †	0.0	*-2.3
cleaved adhesin domain protein, partial	0.0	0.0	*1.4	*-2.0
CoA binding domain protein	0.0	-0.1	*0.8	-0.4
conserved exported hypothetical protein	*0.6†	* 0.4	-0.2	0.0
cytochrome C and Quinol oxidase polypeptide I	0.0	* 0.3	*0.7	0.1
D-alanyl-lipoteichoic acid biosynthesis protein DltB	*0.6	0.0	0.2	0.0
DeoR family transcriptional regulator	0.0	0.1	*0.7	0.7
Dihydrolipoamide dehydrogenase of acetoin dehydrogenase	*-0.5	* -0.5	*-1.1	*1.4
dihydroorotase	*0.5†	* 0.3 †	0.0	*-1.7†
dihydropyrimidine dehydrogenase subunit A	0.0	0.0	0.3	*-1.4
DNA gyrase, B subunit, C-terminal domain protein	*-0.4†	* -0.9	*-1.3	1.4
DNA-directed RNA polymerase subunit beta	*1.2	0.0	0.2	0.0
DNA-directed RNA polymerase subunit beta'	0.0	0.1	*1.0	-0.5
DNA-directed RNA polymerase, beta subunit, partial	*0.2†	0.2	*-1.1	-0.1
DNA-directed RNA polymerase, partial	*0.5	-0.1	*0.6	0.1
dTDP-glucose 4,6-dehydratase	0.0	0.0	0.5	*-2.6
electron transport complex, RnfABCDGE type, A subunit	*-1.1	* -0.7	-0.3	-0.2
F0F1 ATP synthase subunit B	*0.8†	* 0.4	0.0	-0.7
fatty acid-binding protein DegV	0.0	* -0.6	*-1.0	*3.0
flagellar basal body rod protein FlgF	*-0.6†	* -1.0	-0.3	*0.8†
flagellar basal-body rod protein FlgC	0.0	* 0.4	*0.5	*-1.3
flagellar filament outer layer protein Flaa	*-1.3	-0.2	-0.3	0.1
flagellar hook-associated protein 2	0.0	* -1.2 †	0.0	0.0
flagellar hook-associated protein 2 C-terminal domain protein	*-1.4†	0.0	-0.7	*2.6
flagellin B	0.0	* 0.6 †	0.5	*-1.3†
FMN-binding domain protein, partial	*-0.4	* 0.7 †	-0.0	0.5
gamma-aminobutyrate permease	0.0	* 0.5	-0.1	0.0
gliding motility protein Gldk	*0.5	0.1	0.2	0.2
Glucosamine--fructose-6-phosphateaminotransferase	0.0	* -0.6	*-1.5	*1.8
glucose-1-phosphate cytidylyltransferase	*-0.7	* -0.5	*-1.6	*2.9
glycine cleavage T-protein	0.0	0.0	*-1.6	0.0
glycine dehydrogenase (decarboxylating) beta subunit	0.0	* 0.3	*0.8	*-1.5†
glycine dehydrogenase subunit 1	0.0	* 0.6 †	*0.5	0.1
glycosyltransferase, group 4 family	*-0.5	* -1.1	*-0.7	*2.4
glycyl-tRNA synthetase subunit beta, partial	*0.5†	0.1	0.0	-0.3
GTP-binding protein Der	0.0	0.0	0.0	*2.1
hemagglutinin and invasin like cell surface protein	0.0	* 0.6	0.3	0.2
Hemagglutinin protein	*0.2†	0.3	*1.2	*-1.1†
hemin-binding protein 2	*-1.2	* -0.7	-0.6	-0.5
High-affinity Fe2+ transport protein	0.0	0.0	0.0	*-1.6†
hypothetical protein T231_16195	0.0	0.2	*0.7	-0.9
isopropylmalate isomerase	*0.5	* 0.3	0.1	0.2
lysine-2,3-aminomutase	0.0	0.0	0.0	*-1.6
MarR family transcriptional regulator	*0.7†	* 0.4 †	0.3	*-1.5
membrane protein OxaA	*-0.5†	* -0.5 †	*-0.8	*3.1
Methionine adenosyltransferase 2 subunit beta, partial	0.0	0.2	*0.7	-0.6
molecular chaperone GroES	*0.7†	* 0.6 †	*0.8	-0.7
NADH pyrophosphatase	*-0.5	* -0.5	*-2.2	*2.7
NADH:ubiquinone oxidoreductase subunit N, partial	*0.5	0.1	0.1	0.1
NADP transhydrogenase subunit beta, partial	*-0.9	* -0.3 †	-0.0	0.2
oligopeptide transport ATP-binding protein AmiF	*-0.5†	* -0.8	*-0.8	*1.4
outer membrane protein 41	*-0.8	0.0	0.1	0.8
peptide ABC transporter substrate-binding protein	0.0	-0.0	*1.1	-0.9
periplasmic-binding protein-like domain protein	*-1.2†	* -0.8 †	-0.1	-0.1
phenylalanine--tRNA ligase alpha subunit	*-0.5†	* -1.0 †	-0.1	0.1
phosphoglycerate dehydrogenase	0.0	-0.3	*-1.1	*1.4
phosphoglyceromutase	*0.6	0.1	0.1	0.0
polysaccharide biosynthesis protein GtrA	*-0.9†	* -1.1	*-0.9	*2.2
prepilin-type N	*-0.7†	* -1.1 †	*-1.0	*2.5
preprotein translocase subunit SecD	*0.3	0.2	*0.8	0.1
protein-export membrane protein SecD/SecF	*0.5	0.0	0.0	0.0
putative butyryl-CoA:acetate CoA-transferase	*-1.5	* -0.5 †	-0.3	0.6
putative calcium-transporting ATPase	*-0.6†	* -1.0 †	-0.1	0.7
putative immunoreactive antigen PG32	*-2.0	-0.4	-0.7	*3.4
putative phosphoenolpyruvate-protein phosphotransferase, partial	*-0.3†	-0.3	*-1.1	*1.4
putative R-phenyllactate dehydratase, small subunit	*-1.5	* -0.8	-0.5	1.3
Pyruvate formate-lyase 1-activating enzyme	0.0	-0.1	*-0.9	*2.4
Pyruvate-flavodoxin oxidoreductase	0.0	0.0	*-1.0	0.0
recombinase RecU	0.0	* -1.0 †	0.0	0.0
ribonucleoside-diphosphate reductase, class 1b, alpha subunit	*-0.8†	* -0.7	*-0.8	*2.2
RNAase, partial	0.0	0.1	*0.9	*-0.8
S-layer protein	0.0	* 0.6 †	0.3	0.3
SH3 domain protein, partial	0.0	* -0.2 †	-0.1	*-1.3†
sodium:proton antiporter	0.0	-0.2	*0.9	0.0
starch synthase catalytic domain protein	*-0.9	* -0.5	*-0.7	*1.2
succinate-semialdehyde dehydrogenase	0.0	-0.1	-0.0	*-1.5
surface layer protein B	0.0	* 0.7	*0.5	-0.1
toxin-antitoxin system subunit antitoxin	*-0.4†	* -0.9	*-0.7	*2.2†
translation initiation factor IF-2, nonfunctional	0.0	0.2	*-1.2	*1.2
Type II secretory pathway, component PulF	*-1.5	* -0.6 †	-0.1	0.9
type IV pilus secretin PilQ	*0.2†	* 0.7	-0.5	1.0
UDP-N-acetylmuramate--alanine ligase	*0.3	* 0.6 †	0.3	-0.7
v-fos FBJ oncogene homolog, isoform CRA_b	0.0	* -1.0	0.0	0.0
von Willebrand factor type A domain-containing protein	*-0.9	* 0.8 †	0.1	-0.5
YadA protein	0.0	-0.1	0.2	*2.2
YSIRK type signal peptide	0.0	* -1.4	0.0	0.0

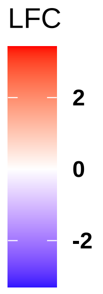

Figure S11 Comparative overview of differentially abundant functions detected across the four groups based on National Center for Biotechnology Information non-redundant (NCBI nr) protein database annotation. Healthy (H), gingivitis (G), diseased periodontitis (DP), and resolved periodontitis (RP) groups were defined based on clinical parameters as described in the Methods section. The numbers "1" and "2" denote pre-treatment and post-treatment samples, respectively. Sample sizes were n = 28 for H, n = 26 for G, n = 13 for DP, and n = 14 for RP. This figure summarizes differentially abundant functions identified by ANCOM-BC2 in longitudinal pre- and post-treatment comparisons within the H, G, DP, and RP groups (H2/H1, G2/G1, DP2/DP1, and RP2/RP1), displayed side by side to facilitate visual comparison of shared and group-specific functional changes across groups. Functions shown in the figure were selected based on statistical significance in at least one comparison (Benjamini–Hochberg-adjusted $q < 0.05$). For these displayed functions, log fold change (LFC) values are shown for all four within-group comparisons, including comparisons that did not reach statistical significance. The color scale represents the LFC between post- and pre-treatment within each group. Asterisks (*) indicate statistically significant comparisons, and functions that additionally passed the ANCOM-BC2 sensitivity analysis are indicated by a dagger symbol (†). Differential abundance analysis was performed using ANCOM-BC2, with q values adjusted for multiple testing using the Benjamini–Hochberg method, and $q < 0.05$ was considered statistically significant. Sensitivity analysis was conducted within ANCOM-BC2 to assess the reliability of the detected results. Although all groups are displayed together for visual comparison, the statistical analyses shown in this figure are exclusively based on longitudinal (paired) comparisons within each group (H1 vs H2, G1 vs G2, DP1 vs DP2, and RP1 vs RP2). Abbreviations: LFC, log fold change; ANCOM-BC2, analysis of compositions of microbiomes with bias correction, version 2; NCBI nr, National Center for Biotechnology Information non-redundant protein database.

Figure S12

	H2/H1	G2/G1	DP2/DP1	RP2/RP1
[CP]ahpC (I917_17140) - alkyl hydroperoxide reductase subunit C	*-2.6	*-2.1†	*-3.3†	*-2.3†
[CP]ahpC (MYCMA_2448) - alkyl hydroperoxide reductase subunit C	-0.4	*-0.5	-0.1	-0.4
[CP]bplD (BB0152) - UDP-N-acetylglucosamine 2-epimerase	*1.4†	0.7	*1.4†	*1.9
[CP]cheY (lse_0602) - chemotaxis response regulator	-0.1	-0.4	-0.7	*-1.3
[CP]ciuD (DIP0585) - Putative iron transport system ATP-binding protein	*-0.6	0.1	-1.0	*-1.0†
[CP]ctpC (MSMEG_6058) - cadmium transporting P-type ATPase	*1.7	-0.2	0.6	-0.0
[CP]ctpV (MUL_0423) - metal cation transporter p-type ATPase, CtpV	-0.2	*-0.6	-0.0	*-1.0
[CP]ctpV (MULP_05109) - metal cation transporter p-type ATPase, CtpV	-0.1	*-0.6	*-1.4	0.2
[CP]fadE29 (MYCMA_0321) - acyl-CoA dehydrogenase fadE12	*1.4	*0.8†	-0.3	0.4
[CP]fagC (cgR_0600) - hypothetical protein	*1.8	*1.7†	*1.4	*1.3†
[CP]flaA (VC0395_A1780) - flagellin core protein A	*0.5†	*1.7	0.1	-0.0
[CP]fliC (Pfl_1527) - Flagellin-like	-0.6	*-0.8	-0.2	*-1.8
[CP]groEL (CAC2703) - chaperonin GroEL	*-0.6	*-0.7	-0.6	-0.2
[CP]groEL (CD0194) - 60 kDa chaperonin	-0.5	*-0.7	-0.2	-0.5
[CP]groEL (CLD_1225) - chaperonin GroEL	-0.0	*-0.8†	-0.5	-0.6
[CP]groEL (CPF_2573) - chaperonin, 60 kDa	-0.2	-0.1	-0.7	*-1.1
[CP]hemH (NTHI1329) - ferrochelatase	-0.1	0.6	0.5	*-2.8
[CP]hisF2 (PA3151) - imidazole glycerol phosphate synthase subunit HisF	*-0.6	0.2	-0.4	0.2
[CP]hitC (CGSHiEE_02795) - iron(III) ABC transporter ATP-binding protein	-0.1	-0.3	*-1.0	0.2
[CP]hspX (Mspyr1_11890) - molecular chaperone	0.1	-0.5	*-0.9	*-1.1
[CP]htpB (LPC_2606) - Hsp60, 60K heat shock protein HtpB	-0.2	-0.1	*-1.1	-0.1
[CP]htpB (lpl0724) - 60 kDa chaperonin (Cpn60)	*-0.6	0.0	0.0	0.0
[CP]icl (MAV_4682) - isocitrate lyase	*1.4	*-1.1	0.0	0.0
[CP]irp6C (DIP0110) - ATP-binding protein Irp6C	*-0.5†	0.3	-0.0	*-1.4
[CP]irtA (J113_09380) - ABC transporter	0.0	0.0	*-0.9	0.0
[CP]irtA (MYCMA_1214) - iron import ATP-binding/permease protein IrtA	*1.2	*1.5	0.8	*1.7
[CP]kasB (JDM601_1649) - 3-oxoacyl-(acyl carrier protein) synthase II	-0.1	*-0.8	-0.5	*-0.9
[CP]kasB (MAP4_1826) - 3-oxoacyl-ACP synthase 2	0.0	-0.2	*-1.6	*-1.3
[CP]kpsF (JJD26997_1778) - arabinose-5-phosphate isomerase	*1.6†	*1.1†	0.5	*1.1
[CP]lap (LMOSLCC2479_1697) - aldehyde-alcohol dehydrogenase	0.0	0.0	0.0	*-1.3†
[CP]lmb (SGO_1314) - metal binding lipoprotein	0.3	0.3	*-0.9†	-0.3
[CP]lpxA (HD1187) - UDP-N-acetylglucosamine acyltransferase	*-0.5	0.1	-0.8	*-1.0†
[CP]lpxC (HSM_0634) - UDP-3-0-acyl N-acetylglucosamine deacetylase	*1.2	*1.2	1.1	*1.7†
[CP]lpxC (NTHI1312) - UDP-3-O-	0.3	*-0.8	-0.0	0.1
[CP]lytR (BT9727_4947) - membrane-bound transcriptional regulator LytR	0.2	-0.0	*-0.9†	-0.4
[CP]mgtB (SARI_03870) - hypothetical protein	*-0.7†	0.4	0.5	0.4
[CP]mip (lpl0829) - macrophage infectivity potentiator	0.0	-0.1	*-1.3	-0.3
[CP]mpa (MUL_2337) - ATPase	0.7	*2.3	*1.7	*2.7
[CP]mprA (BN42_20802) - DNA-binding response regulator	0.0	0.0	*1.4	0.0
[CP]mrsA/glmM (NTHI1664) - predicted phosphomannomutase	0.1	*-0.6	-0.5	*-1.5
[CP]msbA (HI0060) - ABC transporter ATP-binding protein	-0.1	0.3	*-1.0	-0.2
[CP]msrA/B(pilB) (NMA0290) - methionine sulfoxide reductase	0.0	*-0.6	0.0	*-1.4
[CP]mtrD (NGO1364) - antibiotic resistance efflux pump component	*-0.7	*-0.5	0.9	-0.8
[CP]mtrD (NMA1969) - drug efflux protein	0.7	*1.4†	1.2	*2.1
[CP]mtrD (NMAA_1426) - drug efflux transport protein MtrD	0.3	*1.0†	*2.5	*2.0
[CP]narG (MIP_01937) - nitrate reductase subunit alpha	*-0.6†	0.1	-0.2	0.0
[CP]narH (JDM601_1150) - NarH	*0.8†	*1.7	0.6	0.8
[CP]narH (MSMEG_5139) - nitrate reductase, beta subunit	0.7	0.3	*1.7	*1.6
[CP]narI (Mspyr1_15900) - respiratory nitrate reductase subunit gamma	0.6	0.5	*1.3	*1.7
[CP]narI (Mvan_4543) - respiratory nitrate reductase, gamma subunit	*1.4†	*1.6	*2.7†	*1.6†
[CP]ompP2 (HD1435) - outer membrane protein P2 homolog	*-0.9	-0.3	-0.7	0.1
[CP]panD (CCDC5079_3340) - pantothenate kinase	*1.4	*1.8†	0.4	0.4
[CP]pdhB (MHJ_0112) - pyruvate dehydrogenase	*-0.7	-0.2	-0.1	-0.3
[CP]phoP (Mycsm_05696) - response regulator	0.0	*2.1	0.0	0.0
[CP]plr/gapA (SPy0274) - glyceraldehyde-3-phosphate dehydrogenase	*-0.7	0.2	0.3	0.3
[CP]porA (CFF8240_0396) - major outer membrane protein	*0.9	*1.9	1.2	*1.2
[CP]pppA (PSPA7_0146) - probable phosphoprotein phosphatase	0.0	0.0	*1.9	*2.0
[CP]ppsE (JDM601_2211) - polyketide synthase PpsE	*1.4†	0.0	0.0	0.0
[CP]pspA (SPCG_0120) - pneumococcal surface protein A	-0.3	-0.2	*-1.3	-0.5
[CP]pspA (SPH_0232) - PspA	0.6	*1.6	1.5	*4.1
[CP]rmlA (MYCMA_2290) - glucose-1-phosphate thymidylyltransferase	*1.1†	*1.4	*1.6	*1.7†
[CP]rmlA (SSA_1411) - Glucose-1-phosphate thymidylytransferase, putative	0.1	-0.2	*-1.3	-0.5
[CP]rmlA (VF0167) - Glucose-1-phosphate thymidylyltransferase	*1.2	*1.7†	0.6	0.8
[CP]rpoS (lpp1247) - RNA polymerase sigma factor RpoS	*1.1†	*1.0	*1.2	*1.8
[CP]secA2 (Mflv_3408) - SecA DEAD domain protein	*-0.5	-0.1	1.4	-0.7
[CP]sitB (SFV_1378) - Iron transport protein, ATP-binding component	*2.8	*1.8	1.1	*2.1
[CP]sitB (SSON_1751) - Iron transport protein ATP-binding component	0.3	0.7	0.5	*2.0
[CP]slrA (SPH_0870) - peptidyl-prolyl cis-trans isomerase	*-0.7	-0.0	-0.3	-0.2
[CP]sodA (MAP4_3686) - superoxide dismutase SodA	0.5	-0.1	*1.9	*1.6
[CP]speB (M28_Spy1721) - streptococcal pyrogenic exotoxin B	*1.9	*1.8†	*1.5	*2.9
[CP]vapA1 (BH03380) - Virulence-associated protein a	*1.9†	*1.9	*1.2	*1.3
[CP]wbpD (PA3156) - probable acetyltransferase WbpD	-0.3	-0.1	-0.1	*-1.5
[CP]wcbT (BMA2287) - 8-amino-7-oxononanoate synthase	0.0	-0.3	-0.7	*-1.9
[CP]ybtQ (PSPPH_2896) - ABC transporter, ATP-binding/permease protein	-0.3	0.1	-0.7	*-1.1
[CP]YE105_C1500 - Mannose-1-phosphate guanylytransferase, ManC protein	*1.8	*1.4	0.7	*1.7
[CP]zmpB (SPCG_0620) - zinc metalloprotease ZmpB, putative	0.2	0.6	*1.6	*1.5
[VF]fecB - FecB	*0.7	*1.0	*1.7†	*1.1
[VF]flaA - flagellin	-0.0	*-1.0	-1.3	*-1.7
[VF]hlyB - HlyB protein	*1.7	*1.6†	*1.4	*2.3
[VF]hsdR - type I restriction enzyme HsdR, putative	0.5	*2.1†	0.5	*0.8
[VF]mutS - mismatch repair	0.0	0.0	*-0.9	0.0
[VF]ssb - ssDNA-binding protein controls activity of RecBCD nuclease	*-0.6†	-0.3	-0.1	-0.6
[VF]VC1777 - conserved hypothetical protein	-0.1	-0.1	*-1.3	*-1.1
[VF]VC1779 - C4-dicarboxylate-binding periplasmic protein	*1.8	*1.9†	*1.4	0.8
[VF]wcbT - acyl-CoA transferase	0.0	*3.3†	*1.4	*2.2

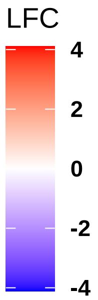

Figure S12 Comparative overview of differentially abundant putative virulence factors detected across the four groups based on Virulence Factors of Pathogenic Bacteria database (VFDB) annotation. Healthy (H), gingivitis (G), diseased periodontitis (DP), and resolved periodontitis (RP) groups were defined based on clinical parameters as described in the Methods section. The numbers “1” and “2” denote pre-treatment and post-treatment samples, respectively. Sample sizes were n = 28 for H, n = 26 for G, n = 13 for DP, and n = 14 for RP. This figure summarizes putative virulence factors identified by ANCOM-BC2 in longitudinal pre- and post-treatment comparisons within the H, G, DP, and RP groups (H2/H1, G2/G1, DP2/DP1, and RP2/RP1), displayed side by side to facilitate visual comparison of shared and group-specific changes across groups. Features shown in the figure were selected based on statistical significance in at least one comparison (Benjamini–Hochberg-adjusted q < 0.05). For these displayed features, log fold change (LFC) values are shown for all four within-group comparisons, including comparisons that did not reach statistical significance. The color scale represents the LFC between post- and pre-treatment within each group. Asterisks (*) indicate statistically significant comparisons, and features that additionally passed the ANCOM-BC2 sensitivity analysis are indicated by a dagger symbol (†). Differential abundance analysis was performed using ANCOM-BC2, with q values adjusted for multiple testing using the Benjamini–Hochberg method, and q < 0.05 was considered statistically significant. Sensitivity analysis was conducted within ANCOM-BC2 to assess the reliability of the detected results. Although all groups are displayed together for visual comparison, the statistical analyses shown in this figure are exclusively based on longitudinal (paired) comparisons within each group (H1 vs H2, G1 vs G2, DP1 vs DP2, and RP1 vs RP2). Features are arranged in alphabetical order. Abbreviations: LFC, log fold change; ANCOM-BC2, analysis of compositions of microbiomes with bias correction, version 2; VFDB, Virulence Factors of Pathogenic Bacteria database.

Figure S13

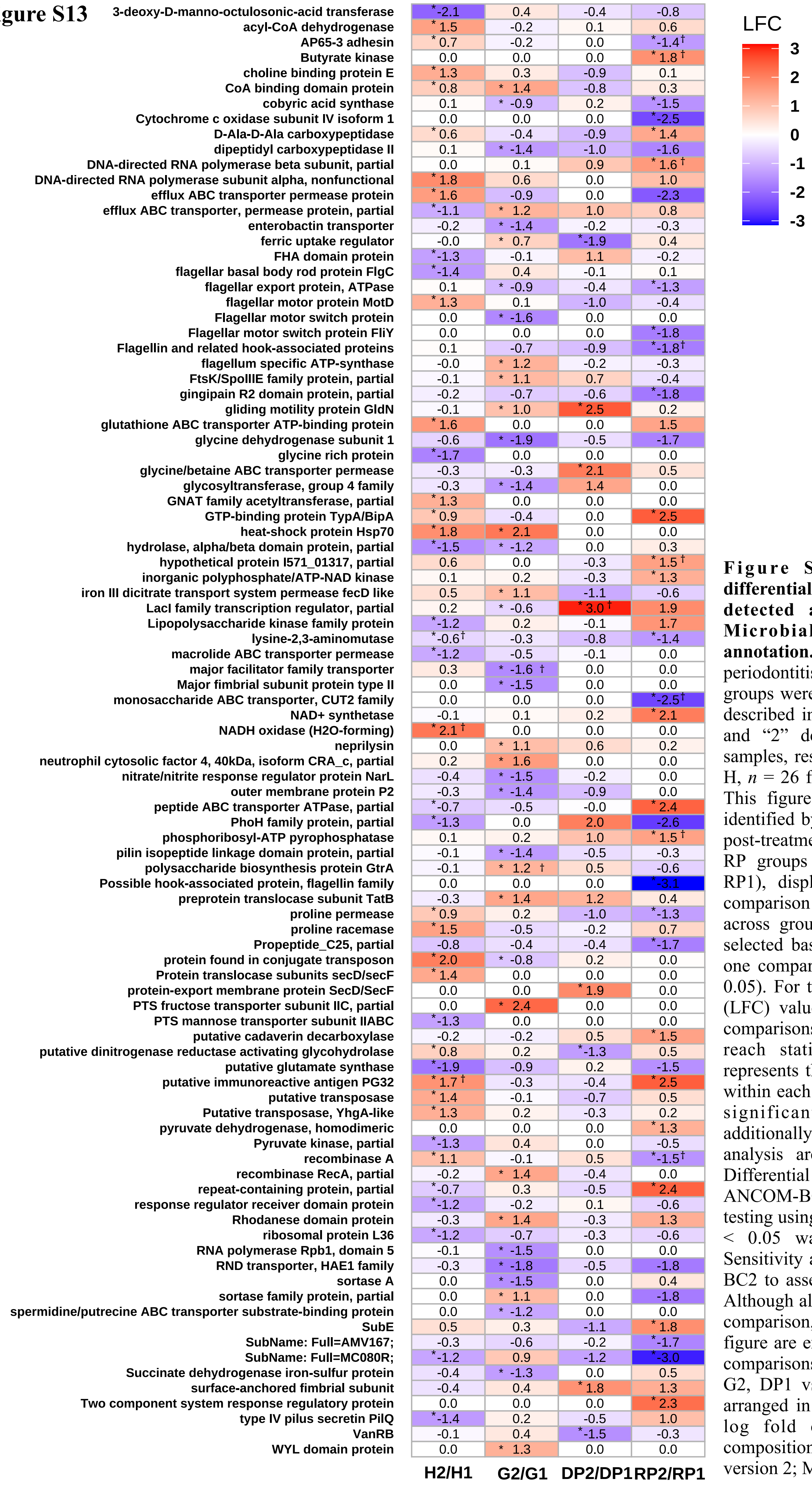

Figure S13 Comparative overview of differentially abundant putative virulence factors detected across the four groups based on Microbial Virulence Database (MvirDB) annotation. Healthy (H), gingivitis (G), diseased periodontitis (DP), and resolved periodontitis (RP) groups were defined based on clinical parameters as described in the Methods section. The numbers “1” and “2” denote pre-treatment and post-treatment samples, respectively. Sample sizes were n = 28 for H, n = 26 for G, n = 13 for DP, and n = 14 for RP. This figure summarizes putative virulence factors identified by ANCOM-BC2 in longitudinal pre- and post-treatment comparisons within the H, G, DP, and RP groups (H2/H1, G2/G1, DP2/DP1, and RP2/RP1), displayed side by side to facilitate visual comparison of shared and group-specific changes across groups. Features shown in the figure were selected based on statistical significance in at least one comparison (Benjamini–Hochberg-adjusted $q < 0.05$). For these displayed features, log fold change (LFC) values are shown for all four within-group comparisons, including comparisons that did not reach statistical significance. The color scale represents the LFC between post- and pre-treatment within each group. Asterisks (*) indicate statistically significant comparisons, and features that additionally passed the ANCOM-BC2 sensitivity analysis are indicated by a dagger symbol (†). Differential abundance analysis was performed using ANCOM-BC2, with q values adjusted for multiple testing using the Benjamini–Hochberg method, and $q < 0.05$ was considered statistically significant. Sensitivity analysis was conducted within ANCOM-BC2 to assess the reliability of the detected results. Although all groups are displayed together for visual comparison, the statistical analyses shown in this figure are exclusively based on longitudinal (paired) comparisons within each group (H1 vs H2, G1 vs G2, DP1 vs DP2, and RP1 vs RP2). Features are arranged in alphabetical order. Abbreviations: LFC, log fold change; ANCOM-BC2, analysis of compositions of microbiomes with bias correction, version 2; MvirDB, Microbial Virulence Database.
